# Six new bacterial species of *Marinilabiliales* isolated from the marine coastal sediment and reclassified *Ancylomarina* and *Labilibaculum* as *Marinifilum* comb. nov. based on the genome analysis

**DOI:** 10.3389/fmicb.2025.1634775

**Published:** 2025-07-23

**Authors:** Han-Zhe Zhang, Jin-Hao Teng, Hao-Yu Zhou, Tian-He Liu, De-Chen Lu, Zong-Jun Du

**Affiliations:** ^1^Marine College, Shandong University, Weihai, China; ^2^Joint Science College, Shandong University, Weihai, China

**Keywords:** *Marinilabiliales*, polysaccharide utilization loci, coastal marine sediments, carbohydrate-active enzymes, genome size, average amino acid identity, percentage of conserved proteins

## Abstract

Evaluation of bacterial succession with cultivation-dependent strategies during enrichment culturing marine sediment led to the isolation of six strains that affiliated with the order *Marinilabiliales*. Six strains were selected for a taxonomic study after discarding clonal cultures. A thorough phylogenetic, genomic and phenotypic analysis of the isolates indicated that they represented six new species. Molecular data revealed the existence of an as yet uncultivated novel species recurrently binned from the enrichment culturing metagenomes. Using a combination of genomic, phylogenetic, and biochemical approaches, we characterized six novel *Marinilabiliales* species capable of degrading marine macroalgal polysaccharides. Bioinformatic polysaccharide utilization loci (PUL) annotations suggest usage of a large array of polysaccharides, including laminarin, α-glucans, and alginate as well as mannans and fucans, highlighting the genus’ involvement in the marine carbon cycle. This study represented a new example of the use of the tandem approach of whole cell mass spectrometry linked to 16S rRNA gene sequencing in order to facilitate the discovery of new taxa by high-throughput cultivation, which increases the probability of finding more than a single isolate for new species. Analysis of CAZymes genes and PUL counts revealed substantial potential for polysaccharide utilization of *Marinilabiliales*. The taxonomic study resulted in the classification of six new species and reclassified *Ancylomarina* and *Labilibaculum* as *Marinifilum* of the order *Marinilabiliales* for which we propose the names *Carboxylicivirga agarovorans* sp. nov., *Carboxylicivirga longa* sp. nov., *Carboxylicivirga caseinilyticus* sp. nov., *Carboxylicivirga litoralis* sp. nov., *Carboxylicivirga fragile* sp. nov., and *Marinifilum sediminis* sp. nov.

## 1 Introduction

Coastal marine sediments represent a highly complex microbial habitat characterized by intense material transformation and energy flow. Despite occupying less than 2% of the global ocean area with an average water depth of less than 50 meters, coastal zones contribute approximately 48% of the global organic carbon flux entering the ocean (Arndt et al., 2013). Polysaccharides, the most structurally diverse organic molecules, dominate marine organic matter and require stepwise microbial degradation involving hydrolysis, fermentation, and respiration processes (Arnosti et al., 2021). Recent advances in microbial ecology have revealed the critical role of the phylum *Bacteroidota* in polysaccharide degradation, particularly in marine systems. Members of the class *Flavobacteriia*, often associated with algal blooms, were key players in breaking down complex carbohydrates derived from marine phytoplankton and macroalgae (Ficko-Blean et al., 2017; Kappelmann et al., 2018; Reisky et al., 2019; Francis et al., 2021). Massive proliferations of these primary producers represent pivotal events in the global carbon cycle, annually removing thousands of megatons of carbon dioxide from the atmosphere and triggering sequential blooms of heterotrophic planktonic bacteria and algae-associated microbes that remineralize much of the fixed carbon (Teeling et al., 2016). *Bacteroidota* were among the primary contributors to this carbon cycling, typically employing polysaccharide utilization loci (PUL)—clusters of adjacent genes encoding carbohydrate-active enzymes (CAZymes) and transport systems—to degrade and assimilate specific polysaccharides (Kappelmann et al., 2018; Francis et al., 2021). However, current research on *Bacteroidota*-mediated polysaccharide degradation has predominantly focused on seawater-associated and bloom-related bacterial species. While a recent study by Sun et al. (2025) has provided valuable insights into the role of *Bacteroidota*, particularly through PULs and chemotaxis-mediated motility, in degrading polymeric carbohydrates within surface sediments of the Pearl River Estuary, our understanding of the broader diversity of major microbial groups involved, their specific substrate preferences, and the complete array of degradation pathways operative across different types of marine sediments and under varying environmental conditions is still evolving. Characterizing these aspects more comprehensively remains crucial to fully understand the complexities of carbon cycling in these diverse and understudied benthic habitats. Notably, the family *Marinifilaceae* emerges as a dominant group in both animal- and plant-derived tissue enrichments, suggesting its ecological versatility in global deep seas (Li et al., 2022). This family’s prevalence may stem from its capacity to degrade complex carbohydrates from diverse organic sources, such as algal exudates, detritus, or animal-derived polymers (e.g., chitin, mucins). For instance, *Ancylomarina* and *Labilibaculum* (*Marinifilaceae*) exhibit variable sulfatase activity, indicating adaptations to sulfated marine substrates, while others, like *Marinifilum*, show specialized CAZyme repertoires for degrading cellulose or hemicellulose from terrestrial inputs (Li et al., 2022).

Studies on *Marinifilales* in nearshore sediments were particularly scarce, leaving gaps in understanding their ecological roles, substrate preferences, and contributions to carbon cycling. Given their prevalence in organic matter-enriched settings, these microbes likely play underappreciated roles in degrading recalcitrant polysaccharides and mediating carbon fluxes. At the time of writing, 4 families, 30 genera and 66 species were assigned to the order *Marinilabiliales*,^1^ including *Carboxylicivirga* (6 species), *Draconibacterium* (5 species), *Marinifilum* (5 species) and *Sunxiuqinia* (5 species). The genus *Carboxylicivirga* and *Marinifilum* both belong to *Marinilabiliaceae*. *Marinilabiliaceae* was a family within the phylum *Bacteroidota* and the largest family in the order *Marinilabiliales*, comprising 26 species. It was primarily distributed in marine sediments, high-salinity environments, or anaerobic ecosystems (Mu et al., 2018). Metabolically diverse *Marinifilaceae* bacteria drive organic matter mineralization across the global deep sea (Li et al., 2022). The genus *Carboxylicivirga* was composed of Gram-stain-negative bacteria that were reported from different marine environments such as tidal flat sediment, sea cucumber culture pond and coastal sediment (Yang et al., 2014). The genus was proposed by Yang et al. (2014) to accommodate two unusual species *Carboxylicivirga mesophila* and *C. taeanensis*. To date, the genus *Carboxylicivirga* includes seven species: *C. mesophila*, *C. taeanensis* (Yang et al., 2014), *C. linearis* (Wang et al., 2015), *C. marina* (Zhang et al., 2021), *C. sediminis* (Wang et al., 2018) and *C. flava* (Wang et al., 2016). Interestingly, cell morphology of *Carboxylicivirga* was slightly curved rod and not all members of the genus *Carboxylicivirga* can degrade agar. The common characteristics within the genus *Carboxylicivirga* were that they were negative for oxidase and H_2_S production and contain MK-7 as the major respiratory quinone. The genus *Marinifilum* was proposed by Na et al. (2009) to accommodate one unusual species. To date, the genus *Marinifilum* includes five species: *M. albidiflavum* (Xu et al., 2016), *M. breve* (Fu et al., 2018), *M. caeruleilacunae* (Dai et al., 2022), *M. flexuosum* (Ruvira et al., 2013) and *M. fragile* (Na et al., 2009).

In the present study we analyzed the cultivable diversity of sediment bacteria of coastal samples. We prioritize genomic and functional analyses of *Marinifilales* to unravel their metabolic strategies and biogeochemical impacts in coastal ecosystems. This study describes the phenotypic, biochemical, and genotypic characterization of strain RSCT41^T^, N1E11^T^, N1Y90^T^, 1640^T^, A049^T^, and A043^T^ concluding that it represents five novel species in the genus *Carboxylicivirga* and one novel species in the genus *Marinifilum*. Here, we present a comparative analysis of polysaccharide utilization loci (PULs) derived from 79 genomes of *Marinilabiliales*, including 55 isolates obtained from marine sediments, resulting in a total of 2,154 manually curated PULs.

## 2 Materials and methods

### 2.1 Strain isolation and culture conditions

We sampled a coastal area in Weihai, China (37°33′36.0″N, 122°07′12.0″E) on October 15th 2018, and in December 2016 from tidal flat located in Yantai, China (37°33′38.7″N, 121°17′7.6″E). Surface sediment (~5 m depth) were collected in triplicate in sterile plastic bags, kept on ice and transported to the laboratory within 2 h. In total, we sequenced four metagenomes and constructed 16S rRNA gene tag libraries, and isolated 757 bacterial strains. The novel strain RSCT41^T^ was isolated from the Jia River, whereas strains N1E11^T^, N1Y90^T^, 1640^T^, A049^T^, and A043^T^ were isolated from sediment collected in Weihai, China. The sediment sample (1 g) was suspended in 9 mL sterile seawater and diluted serially. An aliquot (0.1 mL) from the serial dilution was spread on marine agar 2,216 (MA, Hopebio) and incubated at 28°C for 7 days. Colonies were subsequently purified and used for physiological, biochemical, and chemical analyses. All strains were cultivated on MA at 28°C for 60 h. Cultures were maintained frozen at −80°C in sterile water supplemented with 15% (v/v) glycerol and 1% (w/v) NaCl.

### 2.2 Chemotaxonomic properties

Gram staining and hydrolysis assays (agar, starch, gelatin, alginate, Tween 20, 40, 60, 80) were conducted following Smibert’s et al. (1994) protocols. Colonies grown on MA (33°C, 60 h) were examined for morphology. Cell structure and motility were analyzed via light microscopy (Leica ICC50HD) and phase-contrast microscopy (Nikon E600W), with gliding motility assessed using Bowman (2000) methodologies. DNase activity was tested on DNase Agar (Hopebio) per manufacturer guidelines. Temperature-dependent growth was evaluated across a range of 4–45°C on marine agar (MA), using temperature increments of 5°C or smaller. Growth NaCl tolerance and pH were evaluated as follows: NaCl tolerance: 0–12% (w/v) in modified MB (composition: 5 g tryptone, 1 g yeast extract, 3.2 g MgSO₄, 2.2 g MgCl₂, 1.2 g CaCl₂, 0.7 g KCl, 0.2 g NaHCO₃ per liter), pH tolerance: 5.5–9.5 (adjusted with 20 mM buffers: MES for pH 5.5–6.0; PIPES for 6.5–7.0; HEPES for 7.5–8.0; Tricine for 8.5; CAPSO for 9.0–9.5). To examine O_2_ metabolism, growth under strictly anaerobic condition was tested on MA with or without 0.1% (w/v) NaNO_3_, incubated for 7 days at 33°C. Oxidase activity was determined with an oxidase reagent (bioMérieux). Catalase activity was tested by the observation of gas bubbles after the addition of a few drops of 3% (v/v) H_2_O_2_ to fresh biomass grown on an agar plate. Various biochemical and additional enzyme activities were harvested using API 20E and API ZYM kits (bioMérieux). Oxidation of different compounds as sole carbon sources was checked in Biolog GEN III MicroPlates. All the API kits and Biolog GEN III MicroPlates tests were carried out according to the manufacturer’s instructions except bacterial biomass for inoculations was suspended in 3% (w/v) NaCl.

Cells for fatty acid profiling were cultivated on marine agar (MA) at 28°C for 60 h. Fatty acid extraction and methyl ester analysis were carried out following the protocol established by Sasser (1990), utilizing the MIDI Sherlock Microbial Identification System (Microbial ID Inc.). For isoprenoid quinone characterization, strain RSCT41^T^, N1E11^T^, N1Y90^T^, 1640^T^, A049^T^, and A043^T^ cells were harvested during the late-exponential growth phase in marine broth (MB) at 28°C. Quinones were isolated from lyophilized biomass (300 mg) via chloroform-methanol (2:1, v/v) solvent extraction and purified via high-performance liquid chromatography (HPLC). Polar lipid compositions of strains RSCT41^T^, N1E11^T^, N1Y90^T^, 1640^T^, A049^T^, and A043^T^ were resolved by two-dimensional thin-layer chromatography (2D-TLC). The first direction was developed in chloroform:methanol:water (65:25:4, v/v/v), and the second in chloroform:methanol:acetic acid:water (80:12:15:4, v/v/v/v). Appropriate detection reagents were used to identify the spots; molybdophosphoric acid (phosphomolybdic acid reagent, 5% v/v solution in ethanol; Sigma) was used to detect total polar lipids, ninhydrin reagent (0.2% solution; Sigma) was used to detect amino lipids, Zinzadze reagent (molybdenum blue spray reagent, 1.3%; Sigma) was used to detect phospholipids, and Polar lipids were extracted with different ratio of chloroform/methanol/water system and analyzed by two-dimensional silica thin-layer chromatography according to the modified method described previously (Komagata and Suzuki, 1988).

### 2.3 Whole-genome sequence and phylogenomic analyses

Genomic DNA was isolated from 60-h cultures grown on marine agar (MA) using the TIANamp Bacteria DNA Kit (TIANGEN) according to the manufacturer’s instructions, followed by PCR amplification of the 16S rRNA gene. The genomic DNA G + C content was calculated based on the draft genome sequence, while complete 16S rRNA gene sequences were extracted from individual genomes via Barrnap^2^ and submitted in the GenBank database. Reference strain sequences were acquired from the GenBank/EMBL/DDBJ repositories. 16S rRNA gene sequence similarities were assessed via the EzTaxon-e platform^3^ (Chalita et al., 2024) and BLASTN analysis. Sequence alignment was conducted using CLUSTALW v1.81, with phylogenetic trees reconstructed through neighbor-joining (NJ) algorithms implemented in MEGA v11.0 (Tamura et al., 2021).

### 2.4 Comparative genomic analysis of the order *Marinilabiliales*

The genomic DNA guanine-cytosine (G + C) content was determined via *in silico* analysis of the assembled genome sequence. Quality metrics were assessed using CheckM (Chklovski et al., 2023) to evaluate N50, contig count, completeness, and contamination. Average nucleotide identity (ANI) values were calculated with the pyani (v0.2.8) (Pritchard et al., 2016). To infer taxonomic boundaries at the genus level, the average amino acid identity (AAI) (Rodriguez-R and Konstantinidis, 2014) and percentage of conserved proteins (POCP) (Qin et al., 2014) were employed for prokaryotic genus delineation, with evolutionary relationships further clarified through this metric. Previous studies have validated POCP as a robust genomic indicator for prokaryotic genus classification. Phylogenomic reconstruction was performed using GTDB-Tk (Chaumeil et al., 2020), leveraging a curated set of 120 universal single-copy marker proteins for robust evolutionary inference, *Algibacter mikhailovii* 4-1,052 was selected as the phylogenetic outgroup. Metabolic pathway profiling was performed using the Kyoto Encyclopedia of Genes and Genomes (KEGG) database (Kanehisa et al., 2016). Carbohydrate-active enzymes (CAZymes) in the genomes were annotated using the dbCAN2 meta-server (Zhang et al., 2018), a specialized tool for automated CAZyme characterization. PULs, PUL-like and other CAZyme-rich gene clusters (CGCs) were predicted by searching annotations of CAZymes and the surrounding upstream and downstream genes, using a seven-gene sliding window such that any CAZyme (excluding glycosyl transferases), sulfatase, TBDT, or susD-like genes would be grouped together if they were within ten genes of another such gene (Francis et al., 2021).

### 2.5 Metagenome-assembled genomes, taxonomic inference of MAGs and abundance analysis

A cumulative sequencing output of 1.4 terabase pairs (Tbp) was obtained, averaging 65 gigabase (Gbp) per metagenomic dataset (Lu et al., 2023). Raw reads underwent quality control through BBDuk v35.14^4^ with FastQC v0.11 for validation. Individual sample assemblies were generated using MEGAHIT v1.2.9 (Li et al., 2015), retaining scaffolds ≥2.5 kbp in length. To facilitate downstream analysis, BBMap v38.86 was employed to map reads back to assemblies, generating BAM files with stringent parameters (minimum identity threshold: 0.99; identity filter: 0.97; fast and nodisk modes enabled). Metagenome-assembled genomes (MAGs) were reconstructed through a hybrid binning strategy within the anvi’o v6.2 platform (Eren et al., 2015), integrating outputs from CONCOCT v0.4.0 (Alneberg et al., 2014), MaxBin2 v2.1.1 (Wu et al., 2016), and MetaBAT2 v0.2 (Kang et al., 2019). Consensus bins were refined using DAS Tool v1.1 (Sieber et al., 2018) to optimize genome completeness. MAG nomenclature follows a hierarchical code: Prefix: Sample origin (B: *Saccharina*, L: *Ulva*, H: *Grateloupia*, R: *Gelidium*, S: seawater, N: sediment) Season: Numeric designation (1: autumn, 2: winter, 3: spring, 4: summer) Suffix: Binning algorithm and unique identifier.

### 2.6 Habitat distribution analysis

The 16S high-throughput sequencing data of Microbe Atlas Project (MAP, https://microbeatlas.org/, accessed on 30 July 2023) was used with a 97% sequence similarity threshold. The taxonomy of each operational taxonomic unit (OTU) was identified by search (Rognes et al., 2016) using the silva SSU Ref NR 99138.1 dataset. OTUs with more than 97% 16S rRNA gene sequence similarity to members of the six novel species of family *Marinilabiliaceae*. The habitat distribution of the six novel species was determined by examining the bacterial abundance in global samples.

## 3 Results

### 3.1 Analysis of morphological, physiological, and biochemical characteristics

Colonies of RSCT41^T^, N1E11^T^, N1Y90^T^, 1640^T^, A049^T^, and A043^T^ on MA were smooth and circular. Bacterial cells were rod-shaped and the lengths all exceed 10.0 μm (Supplementary Figure S1). The optimal salinity of six strains was close to the concentration of seawater (2–3%, w/v), and the optimal temperature was around 28°C. Agar and starch hydrolysis were both positive. Additional biochemical/physiological data were given in Supplementary Table S1 and in the species description. As shown in Supplementary Table S2, the common major fatty acids (>5.0%) of strains RSCT41^T^, N1E11^T^, N1Y90^T^, A049^T^, and A043^T^ were iso-C_15:0_ and anteiso-C_15:0_, which was consistent with other *Carboxylicivirga* species. Unlike the five species of *Carboxylicivirga*, the major fatty acids of strain 1640^T^ (*Marinifilum*) were C_16:0_, C_18:1_ *ω6c*, C_18:0_ 10-methyl, TBSA and Summed Feature 3. The major respiratory quinone detected was MK-7, which was characteristic of the genus *Carboxylicivirga*. The major polar lipids were found to be phosphatidylethanolamine (PE) and one unidentified lipid (L) (Supplementary Figure S2) in N1E11^T^ N1Y90^T^ A043^T^, A049^T^, and RSCT41^T^. The major polar lipids of N1Y90^T^ A043^T^, and A049^T^ also include diphosphatidylglycerol (DPG). Major polar lipids of 1640^T^ include PE and DPG.

The six strains (N1E11^T^ N1Y90^T^ A043^T^, A049^T^, RSCT41^T^, and 1640^T^) exhibited distinct metabolic profiles (Supplementary Table S1). The six strains exhibit distinct metabolic functional divergence, reflecting adaptations to diverse ecological niches. Proteolytic and lipolytic specialists (*C. agarovorans*, *C. litoralis*, and *C. caseinilyticus*) dominate in protein- and lipid-rich environments, evidenced by strong trypsin, α-chymotrypsin, and esterase/lipase activities (C4/C8 and C14 lipase in *C. caseinilyticus*), coupled with gelatin hydrolysis (GEL+). These traits align with roles in decomposing organic matter, marine detritus, or dairy waste. In contrast, *C. longa* specializes in carbohydrate metabolism, uniquely expressing α-galactosidase, β-glucuronidase, α-glucosidase, and α-fucosidase, enabling degradation of plant-derived polysaccharides or host mucins. *C. fragile* prioritizes amino acid catabolism through diverse arylamidases (leucine, valine, cystine) and tryptophan deaminase (TRP+), suggesting adaptation to peptide-rich, carbon-limited niches like soil organic layers or wound exudates. Meanwhile, *M. sediminis* displays metabolic inertness, with weak lipase (C14), β-galactosidase, and nitrate reduction (NO_3_), indicative of survival strategies in oligotrophic sediments through minimal energy expenditure (Supplementary Table S3). All strains except 1640^T^ demonstrated starch hydrolysis, while cellulose degradation was restricted to N1E11^T^, and 1640^T^. Tween utilization varied significantly: N1E11 and N1Y90^T^ showed broad capabilities (Tween 20/40/60/80 and 20/40/60/80 respectively), whereas RSCT41^T^ and A049^T^ failed to utilize any Tweens. Proteolytic activity was observed in N1Y90^T^, A043^T^, and A049^T^ through casein hydrolysis, though none displayed gelatinase activity. DNA and agar degradation were exclusive to N1E11^T^ and RSCT41^T^. Oxidase activity was present in N1E11^T^ and 1640^T^ but absent in RSCT41^T^ (data unavailable for others). Catalase activity differed between oxidase-positive strains, being present in N1E11^T^ but absent in 1640^T^. Nitrate reduction occurred in N1E11^T^, RSCT41^T^, and 1640^T^. Notably, N1E11^T^ demonstrated the most versatile metabolism, while A049^T^ showed limited enzymatic capabilities except for starch and casein utilization. Strain 1640^T^ displayed unique combinations of cellulose degradation, Tween 60/80 utilization, and nitrate reduction despite lacking starch hydrolysis capabilities. These metabolic distinctions suggest ecological specialization and potential differences in environmental adaptation among the strains.

### 3.2 Genome and 16S rRNA gene phylogenies

**Figure 1:**
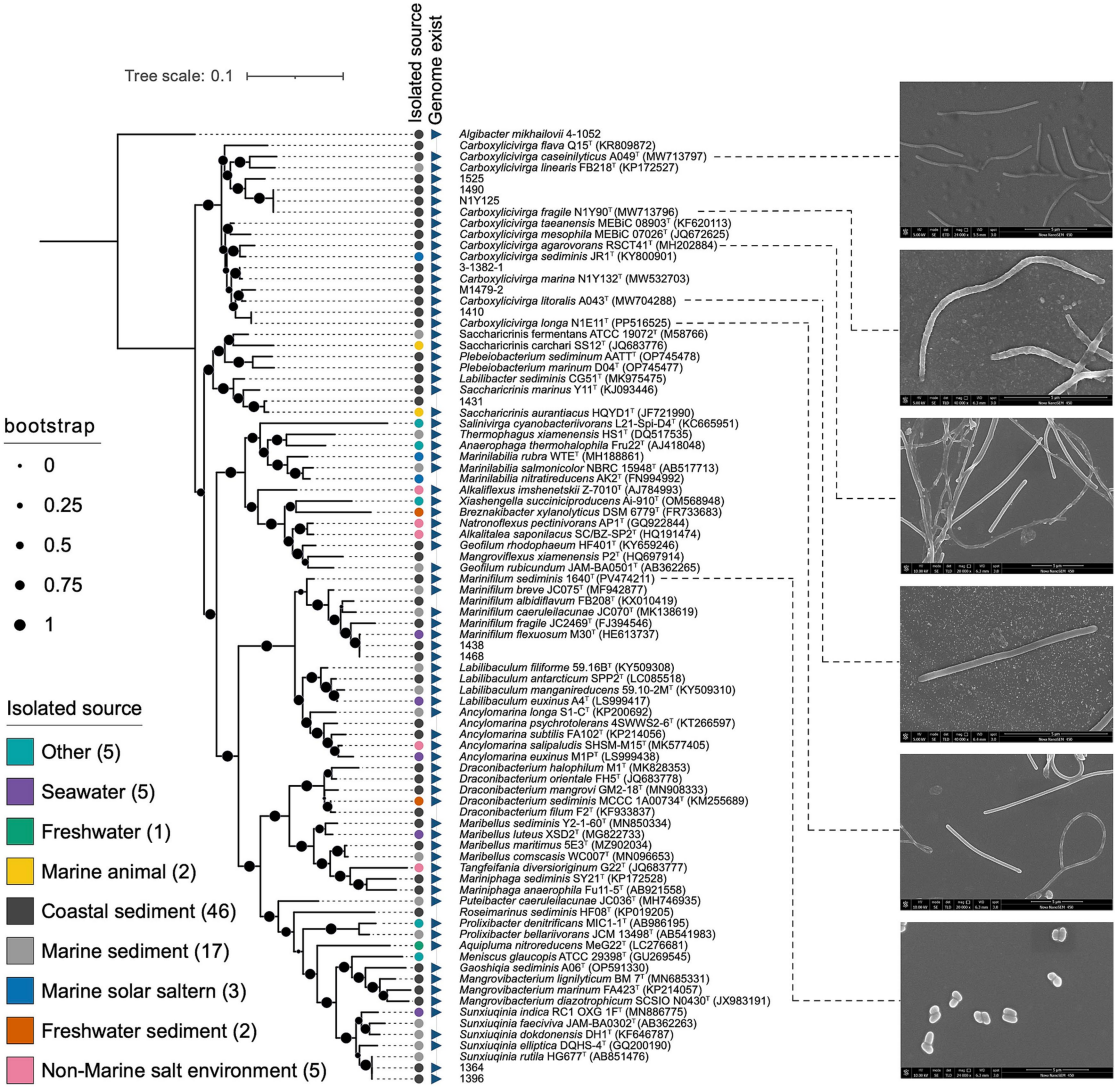
Neighbor-joining phylogenetic tree based on 16S rRNA gene sequences showing the relationship of strains RSCT41^T^, N1E11^T^, N1Y90^T^, 1640^T^, A049^T^, and A043^T^ to members of the order *Marinilabiliales*. Bootstrap values (expressed as percentages of 1,000 replications) of >50% were shown at branching nodes.

The complete 16S rRNA gene sequences of strain N1E11^T^, N1Y90^T^, A043^T^, A049^T^, RSCT41^T^, and 1640^T^ were obtained. Analysis of the 16S rRNA gene sequence revealed that strain N1E11^T^ N1Y90^T^ A043^T^, A049^T^, and RSCT41^T^ belonged to the genus *Carboxylicivirga*, showing the highest sequence similarity with 97.2% similarity to *C. marina* N1Y132^T^, 95.2% similarity to *C. sediminis* JR1^T^, 98.2% similarity to *C. sediminis* JR1^T^, 98.2% similarity to *C. sediminis* JR1^T^, and 97.9% similarity to *C. sediminis* JR1^T^, respectively, 1640^T^ belong to the genus *Marinifilum* showing the highest sequence similarity with 95.9% similarity to *Marinifilum breve* JC075^T^ within the order *Marinilabiliales*. In the neighbor-joining (NJ) phylogenetic tree, strain RSCT41^T^, N1E11^T^, N1Y90^T^, 1640^T^, A049^T^, and A043^T^ formed a distinct clade within the genus *Carboxylicivirga* and *Marinifilum*. Within this subcluster, strain RSCT41^T^, N1E11^T^, N1Y90^T^, A049^T^, and A043^T^ were grouped together with *Carboxylicivirga* species, which was supported by high bootstrap values (Figure 1) and 1640^T^ was grouped together with *Marinifilum breve* JC075^T^.

The order *Marinilabiliales* was ubiquitous in marine sediment ecosystems, playing critical roles in organic matter decomposition and nutrient cycling. The family *Marinilabiliaceae* (*n* = 45) was predominantly found in coastal sediments (71%), followed by marine sediments (22%), and rarely detected in non-marine environments (7%). *Prolixibacteraceae* (*n* = 15) exhibited a broad distribution, with the highest prevalence in coastal sediments (53%) and marine sediments (33%). *Marinifilaceae* (*n* = 8) specialized in marine sediments (75%) and seawater (25%). *Salinivirgaceae* (*n* = 3) exclusively associated with “Other” habitats (e.g., non-marine salt environments). This study aggregates genomic data from diverse environments (coastal sediment, marine sediment, seawater, etc.) to explore how genomic features vary across genera and correlate with environmental adaptation.

### 3.3 Phylogenetics, ANI, AAI, POCP clustering, phylogenomics, and population genomics

**Figure 2:**
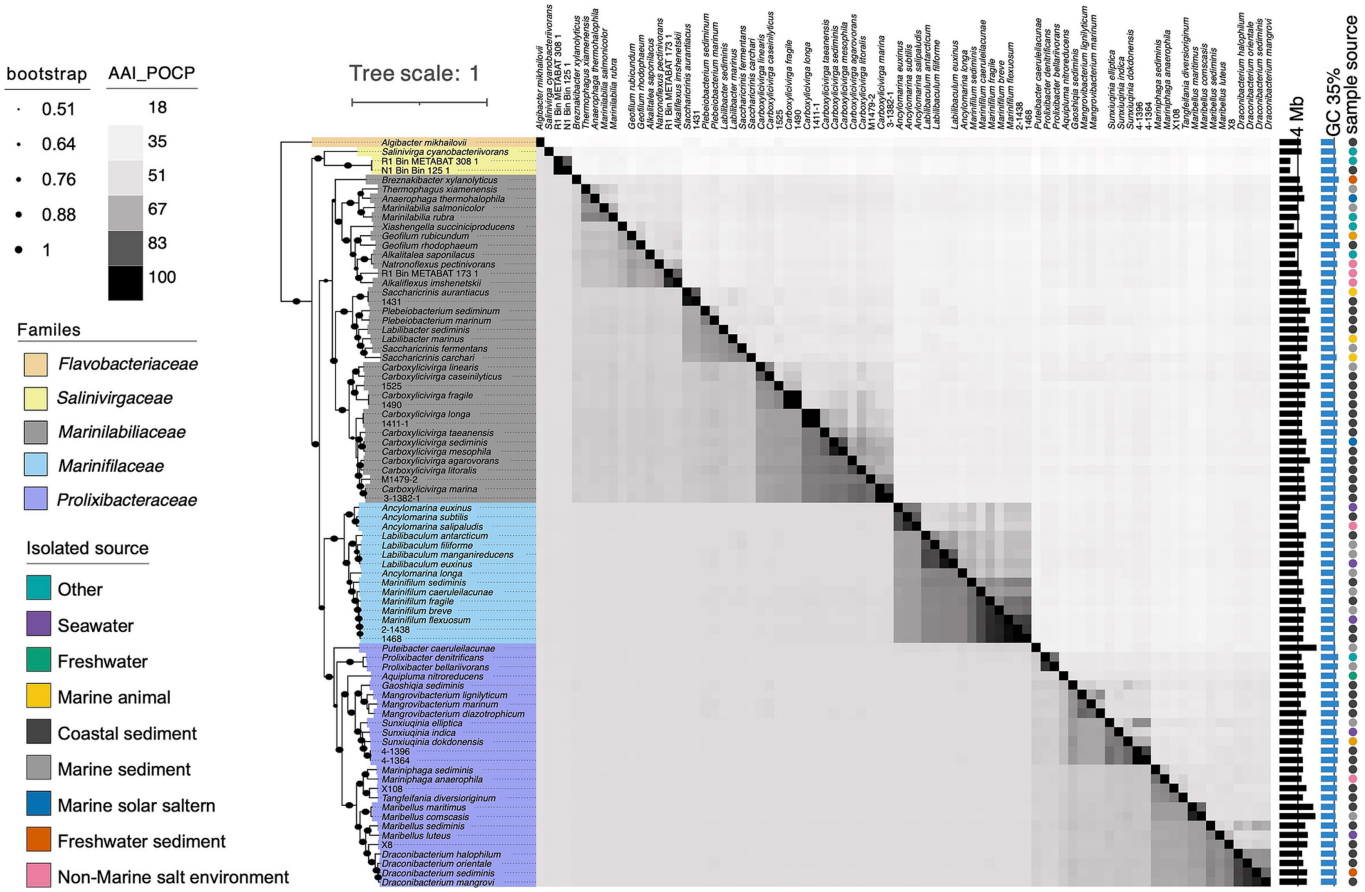
Heatmap comparing average amino acid identity (AAI) and percentage of conserved proteins (POCP) among strains RSCT41^T^, N1E11^T^, N1Y90^T^, 1640^T^, A049^T^, and A043^T^, other members of the order *Marinilabiliales*, and three metagenome-assembled genomes (MAGs) derived from macroalgal surfaces and marine sediments. The lower-left triangle shows AAI values, while the upper-right triangle displays POCP values. Color intensity reflects similarity levels, with darker shades representing higher values. Associated genome sizes (Mb), G + C contents (%), and isolation sources are also indicated.

Phylogenetic reconstructions showed consistent topologies regardless of the sequences used to reconstruct them. Specifically, (i) the 16S rRNA genes (Figure 1), (ii) the concatenated sequences of 120 conserved single-copy orthologous genes (essential genes; Figure 2). The general features of the genomes were given in Supplementary Table S4. The phylogenomic tree can be roughly divided into 4 families. The largest family was relatively small and contains 58 type strains. It represents a highly diverse group in terms of genomic size and ecological diversity, with strains from various environments. Overall, this division provides valuable insights into the evolutionary, ecological, and genomic diversity among these strains, highlighting the differences in model species representation and the variability in genomic characteristics across difference families. A detailed summary of the overall genome-relatedness indices was provided in Supplementary Table S4. The inconsistencies between the phylogenetic trees based on the 16S rRNA gene sequences and the phylogenomic trees constructed from whole-genome sequence analyses reveal that 16S rRNA gene sequence analyses were insufficient to understand the phylogeny and evolution of the members of the genus *Carboxylicivirga*. Both the sequence similarities and phylogenetic relationships indicated that strains RSCT41^T^, N1E11^T^, N1Y90^T^, A049^T^, and A043^T^ represent five novel species of the genus *Carboxylicivirga*.

#### 3.3.1 POCP and AAI analysis of five *Carboxylicivirga* species

The average POCP values between strains RSCT41^T^, N1E11^T^, N1Y90^T^, A049^T^, and A043^T^ with other members of the genus *Carboxylicivirga* were 63.5% (Figure 2 and Supplementary Table S5), exceeding the established genus-level threshold of 50–60%. The analysis of average POCP values among the selected strains (RSCT41^T^, N1E11^T^, N1Y90^T^, A049^T^, and A043^T^) reveals distinct patterns of genomic divergence and potential taxonomic relationships within the genus *Carboxylicivirga*. Strain A049^T^ demonstrated moderate divergence, with AAI values ranging from 53.52% (against *Carboxylicivirga mesophila*) to 63.15% (against 1,490), yielding a mean of 58.34%. This broad range suggests significant genetic differences from several compared strains, particularly *C. mesophila*. In contrast, N1Y90^T^ exhibits extreme variability: its AAI reaches 99.6% with strain 1,490—a value indicative of potential conspecificity—while dropping to 58.66% against *Carboxylicivirga marina*, resulting in a mean of 63.01%. This stark contrast highlights the need to validate the high AAI pair (N1Y90^T^-1490) for potential taxonomic overlap or methodological artifacts. RSCT41^T^ shows moderate divergence overall, with POCP values spanning 54.38% (against *C. linearis*) to 69.92% (against *C. mesophila*), averaging 63.14%. The highest identity with *C. mesophila* suggests closer evolutionary ties to this species. N1E11^T^, on the other hand, displays a remarkable 99.58% POCP with strain 1,411-1, strongly implying genomic similarity that may warrant reclassification, while its lowest identity (54.40% with 1,525) underscores divergence from other lineages. Its mean AAI of 63.26% aligns with broader trends of moderate conservation across the group. A043^T^ exhibits the highest POCP value (70.11%) against strain M1479-2, suggesting a closer genetic relationship, though its mean AAI of 63.24% and minimum value of 56.31% (against *C. linearis*) reflect overall divergence typical of distinct species.

Strain A049^T^ demonstrated moderate AAI values, averaging 71.02%, with a maximum of 82.6% against *Carboxylicivirga linearis* and a minimum of 69.91% against M1479-2 (Figure 2 and Supplementary Table S5). The elevated identity with *C. linearis* suggests potential evolutionary proximity to this species, while lower values (~70%) against most other strains, such as N1E11^T^ (70.43%) and *C. marina* (70.09%), align with its classification as a distinct species. N1Y90^T^ exhibits striking variability, with a 100% AAI value against 1,490—consistent with its previously observed 99.6% AAI—strongly supporting conspecificity. However, its AAI values drop sharply to 70–75% against other strains (e.g., 70.56% with 1,411-1 and 71.11% with *C. marina*), reflecting broader genus-level divergence. RSCT41^T^ shows a moderate mean AAI of 75.12%, ranging from 69.68% (vs. *C. linearis*) to 84.93% (vs. A043^T^). The peak value with A043^T^ highlights a closer functional relationship between these strains, though values below 85% suggest they remain distinct at the species level. Similarly, N1E11^T^ displays a 100% AAI with strain 1,411-1, mirroring its 99.58% AAI, which strongly indicates taxonomic overlap. Outside this pair, N1E11^T^’s AAI values range from 69.82% (vs. *C. linearis*) to 77% (vs. *C. sediminis*), consistent with its divergence from other lineages. A043 exhibits the highest mean AAI (76.73%), peaking at 84.93% with RSCT41^T^—a value approaching genus-level thresholds—while maintaining lower identities (70–79%) with most strains, such as 70.66% against *C. linearis* and 78.4% with M1479-2. This pattern suggests conserved functional traits with RSCT41^T^ but broader divergence across the genus.

#### 3.3.2 AAI and POCP analysis of *Marinifilaceae* reclassified *Ancylomarina* and *Labilibaculum* as *Marinifilum* comb. nov. based on the genome analysis

The novel species 1640^T^ exhibits distinct genomic divergence from other species within the genus *Marinifilum*, as indicated by average amino acid identity (AAI) values. Comparative analysis revealed that strain 1640^T^ shares 79.7–80.2% AAI with validated species in the genus, including *M. caeruleilacunae* (80.0%), *M. fragile* (80.16%), *M. breve* (79.73%), and *M. flexuosum* (79.99–80.09%). These values were significantly lower than the AAI thresholds typically used for species delineation (≥95%), suggesting that 1640^T^ represents a distinct species within *Marinifilum*. In contrast, closely related species such as *M. breve* and *M. flexuosum* share much higher AAI values (91.52–98.61%) among themselves, indicating tight genomic conservation consistent with intraspecific or conspecific relationships. For example, strains of *M. flexuosum* (e.g., 2-1,438 and 1,468) exhibit 97.54–98.61% AAI, strongly supporting their classification as the same species. The relatively low AAI values between 1640^T^ and other *Marinifilum* species (<81%) highlights its genetic distinctness. This divergence aligns with its proposed status as a novel species. However, the AAI range (~80%) is consistent with membership in the same genus, as interspecies AAI values for *Marinifilum* generally fall between 70–95%. The percentage of POCP analysis for the novel species 1640^T^ reveals distinct functional divergence from other validated species within the genus *Marinifilum*. 1640^T^ exhibits POCP values ranging from 71.44% (vs. *M. caeruleilacunae*) to 76.71% (vs. 1,468), with a mean of 74.93%. These values were consistently lower than the intraspecific POCP thresholds observed among closely related species in the genus. *M. breve* and *M. flexuosum* show moderate conservation (78.19–79.53%), while *M. fragile* and *M. flexuosum* exhibit higher POCP values (79.07–80.10%), reflecting their closer evolutionary ties. In contrast, 1640^T^’s POCP values (71.44–76.71%) fall below the typical genus-level threshold of ~85–90%, reinforcing its status as a novel species. This pattern aligns with its previously reported AAI values (79.7–80.2%), which also fall short of species-level thresholds (≥95%). Comparative analysis suggests that 1640^T^’s conserved protein profiles were distinct from those of established *Marinifilum* species, yet consistent with genus membership. The observed POCP range (71–77%) overlaps with interspecific values within the genus (e.g., *M. caeruleilacunae* vs. *M. fragile*: 71.69%; *M. breve* vs. *M. flexuosum*: 78.22%), further supporting its classification as a novel species within *Marinifilum*.

**Figure 3:**
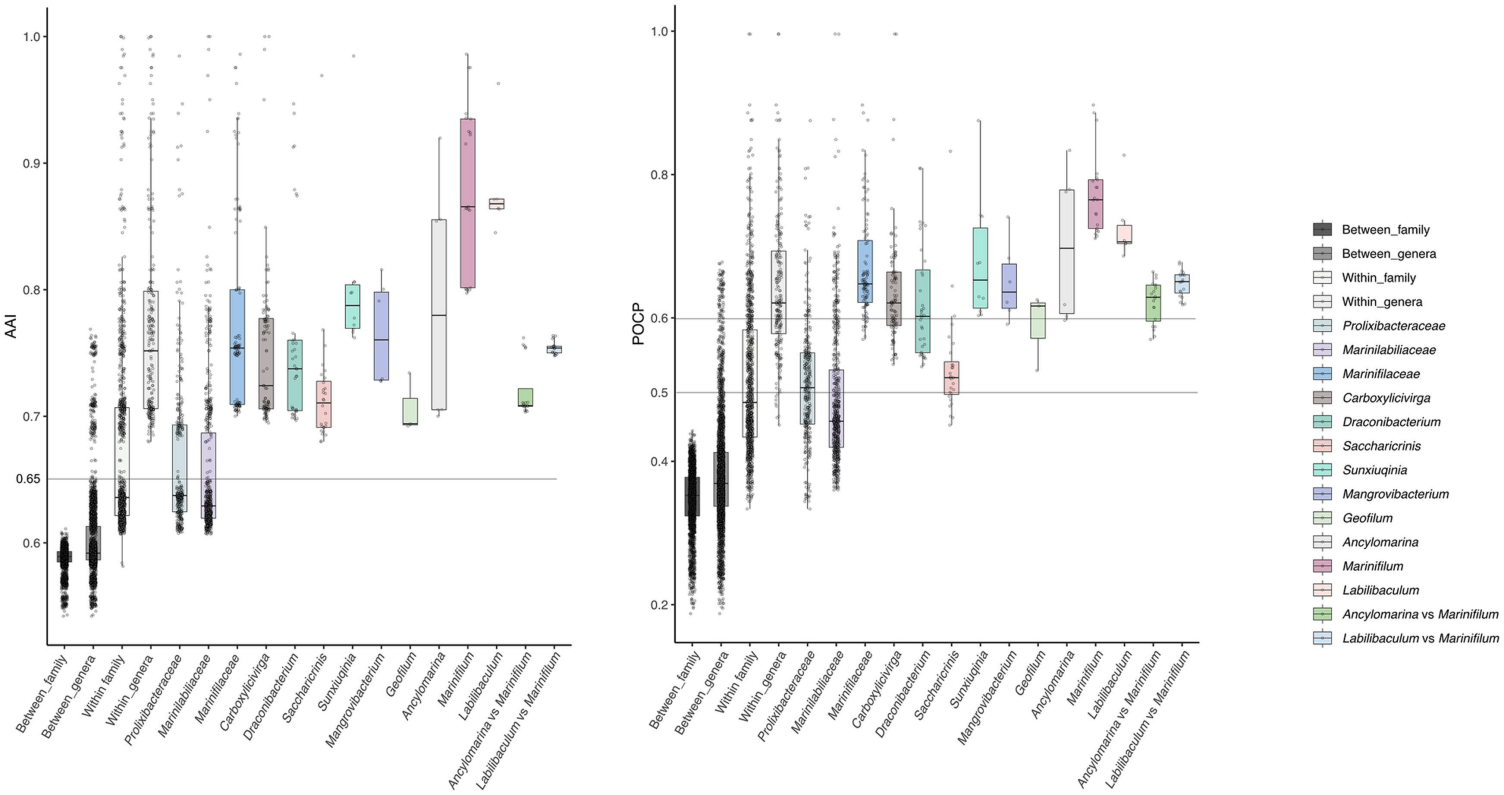
The distribution of AAI and POCP within and between genera was shown in the plots. Histograms at the top of each plot display the distribution of all POCP values, while histograms on the right side show the distribution of all AAI values. Horizontal lines on the plots represent the proposed AAI and POCP threshold values for delimiting genera (refer to the text for further details).

The average amino acid identity (AAI) values between *Ancylomarina* and *Marinifilum* (mean = 72.0%, range: 70.34–76.2%) (Figure 3 and Supplementary Table S5) and between *Labilibaculum* and *Marinifilum* (mean = 75.4%, range: 74.78–76.38%) exceed the proposed genus-level threshold of 65% (Oren and Garrity, 2018). These results suggest that both *Ancylomarina* and *Labilibaculum* fall within the same genus as *Marinifilum* under this criterion. Notably, the AAI ranges for these inter-genus comparisons are significantly higher than the overall between-genus average (60.2%) and align with the lower bounds of intra-genus variability observed for *Marinifilum* (79.73–98.61%) and *Ancylomarina* (67.99–91.98%). The intra-genus AAI distribution of *Ancylomarina* reveals a lower bound (67.99%) that approaches the 65% threshold, indicating potential taxonomic fluidity within this group. This observation further supports the integration of *Ancylomarina* into the *Marinifilum* genus, as its inter-genus AAI values with *Marinifilum* (70.34–76.2%) are comparable to its intra-genus variability. Similarly, *Labilibaculum* exhibits strong coherence with *Marinifilum*, with inter-genus AAI values (74.78–76.38%) exceeding the 65% threshold by a margin greater than that of *Ancylomarina*. These findings align with the proposed AAI-based genus delineation framework, where organisms sharing ≥65% AAI are considered congeneric. The percentage of conserved proteins (POCP) values between *Ancylomarina* and *Marinifilum* (mean = 62.2%, range: 57.0–66.4%) (Figure 3 and Supplementary Table S5) and between *Labilibaculum* and *Marinifilum* (mean = 64.8%, range: 61.9–67.8%) align with proposed genus-level thresholds for bacterial classification (≥50% POCP), providing genomic evidence to support their reclassification into the *Marinifilum* genus. These inter-genus comparisons exceed the overall between-genus average (38.1%) and approach the lower bounds of intra-genus variability observed for *Marinifilum* (71.1–89.7%), though they fall short of the intra-genus thresholds for *Ancylomarina* (59.7–83.4%) and *Labilibaculum* (68.6–82.7%). Notably, the intra-genus POCP distribution for *Ancylomarina* reveals a low boundary (59.7%) overlapping with its inter-genus values, suggesting taxonomic fluidity within this group. The *Labilibaculum*–*Marinifilum* comparison, however, shows stronger coherence (64.8%) and aligns more closely with the lower limit of *Marinifilum*’s intra-genus variability. These findings mirror prior AAI trends, where both genera exhibited intermediate similarity to *Marinifilum* relative to intra- and inter-genus baselines, reinforcing the consistency of genomic metrics across independent analytical frameworks. While POCP alone cannot dictate taxonomic revisions, its convergence with AAI data strengthens the case for merging *Ancylomarina* and *Labilibaculum* into *Marinifilum*. In conclusion, the AAI and POCP metrics presented here provide robust evidence for merging *Ancylomarina* and Labilibaculum into the *Marinifilum* genus under the 65% and 50–60% threshold criterion. This reclassification would streamline taxonomic consistency within this clade while highlighting the need for integrative approaches to validate genus boundaries in light of genomic data.

### 3.4 Habitat and global geographical distribution of the species of five *Carboxylicivirga* sp. and one *Marinifilum* sp. nov.

The dataset exhibits substantial variability in sample sizes across strains and habitat categories, which may influence the reliability and interpretation of the observed ecological patterns (Supplementary Figure S2 and Supplementary Table S6). For instance, *Carboxylicivirga* strains A049^T^, N1E11^T^, and A043^T^ collectively account for thousands of samples in major categories (e.g., *n* = 9,626 for unknown aquatic habitats), whereas strain N1Y90 has a notably smaller total sample size (*n* = 98). Among *Carboxylicivirga* strains, a genus-level consistency was observed for lineages A049^T^, N1E11^T^, and A043^T^, all displaying uniform sediment-associated abundances (8.43%, *n* = 3,392) despite their primary dominance in seawater (~16.4%, *n* = 6,610) and unknown aquatic habitats (23.9%, *n* = 9,626) (Supplementary Figure S2 and Supplementary Table S6). Notably, these strains also shared significant animal associations (~11.9%, *n* = 4,795), suggesting a dual ecological strategy favoring both aquatic and host-linked niches. Strain N1Y90^T^ deviated from this pattern, exhibiting an elevated sediment affinity (10.2%, *n* = 10) within a limited total sample size (*n* = 98), alongside a disproportionate representation in unknown aquatic habitats (26.5%, *n* = 26). This anomaly may reflect either localized adaptation to sedimentary conditions or sampling artifacts, necessitating further validation. In contrast, strain RSCT41^T^ demonstrated minimal sediment colonization (4.02%, *n* = 18), instead thriving in poorly characterized aquatic environments (35.9%, *n* = 161) and marine habitats (19.86%, *n* = 89), with negligible presence in plant rhizospheres (0.22%, *n* = 1). Comparative analysis revealed genus-specific divergence in sediment utilization. While *Carboxylicivirga* strains collectively exhibited stronger sedimentary associations (~8.43%) compared to *Marinifilum* (7.1%), intra-genus variability was evident. For instance, N1Y90^T^’s higher sediment prevalence (10.2%) contrasted sharply with RSCT41^T^’s low abundance (4.02%), underscoring strain-level adaptability to environmental heterogeneity. These differences may arise from metabolic plasticity or uncharacterized habitat gradients. However, the substantial proportion of “unknown” habitat classifications across all strains (e.g., 29.3% for *Carboxylicivirga*, *n* = 11,800) indicates critical gaps in environmental sampling, particularly for sedimentary systems. Similarly, *Marinifilum* (1640) shows a large sample pool (*n* = 7,808), but terrestrial niches like soil (*n* = 208) and plant-associated samples (*n* = 115) were underrepresented (Supplementary Figure S2 and Supplementary Table S6). The examined bacterial strains exhibited distinct ecological preferences, with sediment environments serving as a secondary but notable niche across most lineages. *Marinifilum* (Strain 1,640), predominantly associated with aquatic habitats, showed moderate sediment colonization (7.1%, *n* = 555), ranking below its prevalence in seawater (23.85%, *n* = 1858) and unknown aquatic environments (30.7%, *n* = 2,401). In contrast, terrestrial niches such as soil (2.66%, *n* = 208) and plant-associated samples (1.48%, *n* = 115) were minimally represented, while animal-derived samples accounted for a minor fraction (4.45%, *n* = 349).

In conclusion, sediment environments represent a stable yet subordinate niche for most *Carboxylicivirga* lineages, complementing their dominance in open-water habitats. *Marinifilum*’s intermediate sediment affinity aligns with its broader aquatic versatility. Future studies should prioritize expanded sampling of sedimentary ecosystems, especially for low-abundance strains like RSCT41^T^ and N1Y90^T^, to unravel their ecological roles and adaptive mechanisms in these understudied environments.

### 3.5 Metabolic pathways analysis

**Figure 4:**
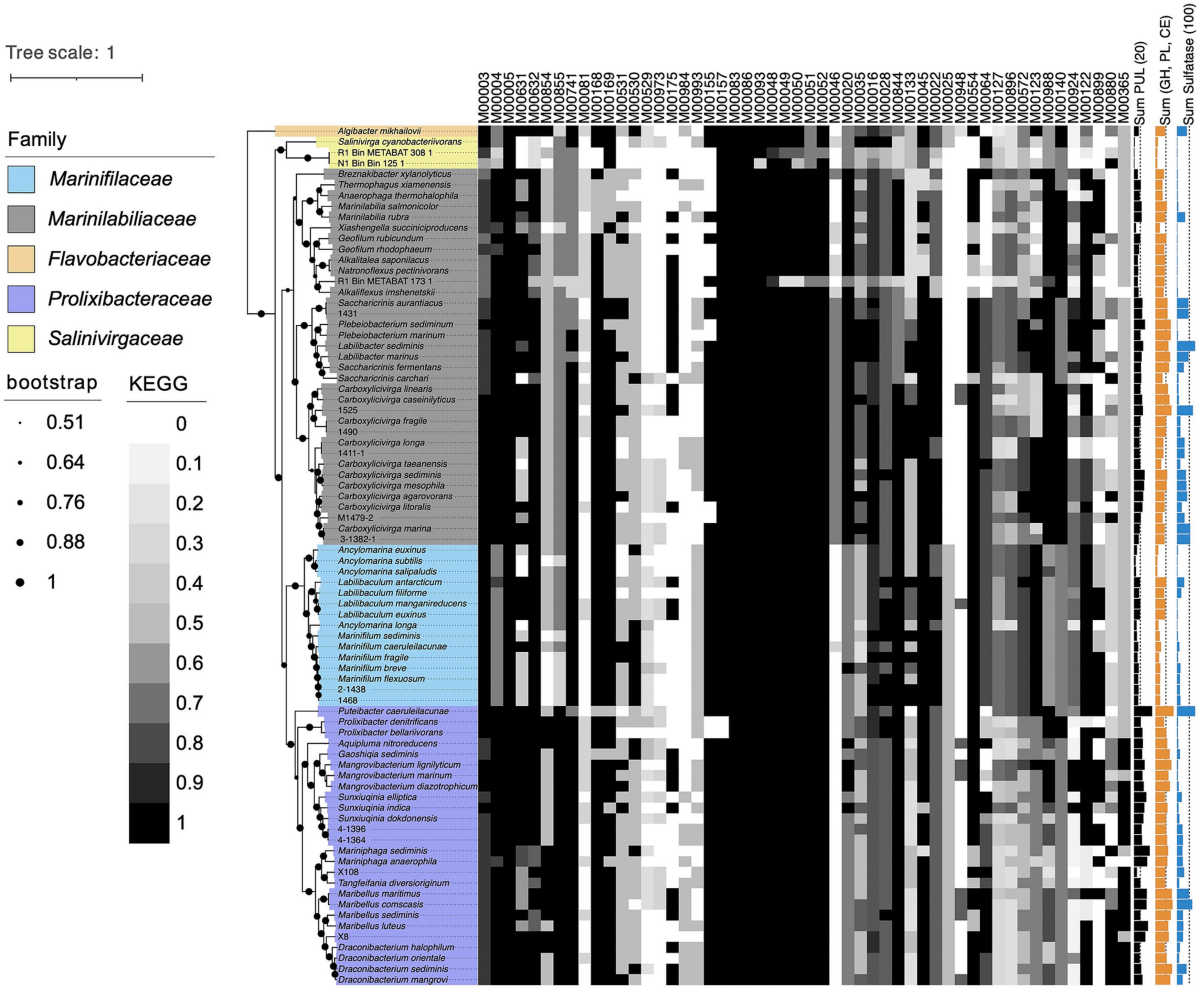
Taxonomic classification of strains RSCT41^T^, N1E11^T^, N1Y90^T^, 1640^T^, A049^T^, and A043^T^ based on the topological structure of phylogenomic tree. Different colors were used to represent different clades. Black filled circles indicates that the bootstrap values were greater than 50.0%, and gray filled circle indicates bootstrap values between 50–100%. Bar, 0.1 substitutions per nucleotide position. Heat map shows metabolic pathways in the genomes of different bacteria. The shade of the color indicates the integrity of the metabolic pathway, with dark gray indicating an intact metabolic pathway. In addition, different shapes, such as circles, squares and triangles, show the genome type. Sum of PUL, CAZymes gene and sulfatase genes of different bacterial species were represented by the bar graphs on the right. Circles of different colors represent different environmental sources.

Firstly, we analyzed the similarities and differences in metabolic pathways between strains RSCT41^T^, N1E11^T^, N1Y90^T^, 1640^T^, A049^T^ and A043^T^ and their closely related strains of the order *Marinilabiliales* (Figure 4 and Supplementary Table S7). Summary of metabolic pathway completeness at the genus level: The provided KEGG analysis data were grouped at the genus level to assess the completeness of metabolic pathways across taxa. Each value represents the percentage completeness of a specific pathway module for members of a genus, averaged across all species within that genus. The following section summarizes key findings, organized by metabolic category and representative genera. (1) Carbohydrate metabolism key genera: *Saccharicrinis*: Exhibited high completeness in central carbohydrate metabolism (average 95–100%), including gluconeogenesis (M00003: 92%) and the pentose phosphate pathway (M00004: 100%). Notably, glycogen biosynthesis (M00854: 83%) and degradation (M00855: 90%) were well conserved. *Carboxylicivirga*: Demonstrated robust pectin degradation (M00081: 85%) and galactose metabolism (M00632: 95%), suggesting adaptation to complex carbohydrate utilization (Figure 4 and Supplementary Table S7). *Marinilabilia*: Showed variability in “other carbohydrate metabolism” pathways (e.g., D-galacturonate degradation: 60–80%), potentially reflecting niche-specific adaptations. (2) Energy metabolism key genera: *Ancylomarina*: High completeness in nitrogen fixation (M00175: 75%) and sulfur oxidation (M00984: 90%), indicative of roles in anaerobic environments. *Draconibacterium*: Dominated dissimilatory nitrate reduction (M00530: 85%) and denitrification (M00529: 70%), aligning with their prevalence in nitrate-rich sediments. *Geofilum*: Displayed near-complete carbon fixation pathways (CAM pathways: 95–100%), suggesting phototrophic or chemolithoautotrophic capabilities (Figure 4 and Supplementary Table S7). (3) Lipid metabolism key genera: *Labilibaculum*: Fatty acid biosynthesis (M00083: 98%) and β-oxidation (M00086: 95%) were highly conserved, consistent with lipid-rich marine habitats. *Marinifilum*: Phosphatidylethanolamine biosynthesis (M00093: 90%) was prevalent, likely supporting membrane stability in fluctuating salinity conditions. (4) Amino acid metabolism key genera: *Natronoflexus*: Showed completeness in arginine biosynthesis (M00844: 85%) and histidine degradation (M00045: 75%), critical for osmoregulation in hypersaline environments. *Prolixibacter*: High scores in aromatic amino acid metabolism (M00022: 80%) and polyamine biosynthesis (M00133: 70%), potentially linked to stress response mechanisms (Figure 4 and Supplementary Table S7). (5) Cofactor and vitamin metabolism key genera: *Sunxiuqinia*: Thiamine biosynthesis pathways (M00127: 65–80%) were prominent, essential for enzymatic cofactor provision in nutrient-limited settings. *Mangrovibacterium*: Cobalamin biosynthesis (M00924: 75%) and molybdenum cofactor pathways (M00880: 60%) highlighted their role in redox reactions (Figure 4 and Supplementary Table S7). (6) Terpenoid and polyketide biosynthesis key genera: *Carboxylicivirga*: Terpenoid backbone biosynthesis (M00365: 55–70%) was moderately conserved, suggesting limited secondary metabolite production. *Ancylomarina*: Low scores in this category (<30%) indicate a metabolic focus on core pathways rather than specialized biosynthesis (Figure 4 and Supplementary Table S7). *Saccharicrinis* and *Carboxylicivirga* dominated carbohydrate metabolism, likely facilitating decomposition of organic matter in marine ecosystems. *Draconibacterium* and *Ancylomarina* specialized in nitrogen and sulfur cycling, aligning with their prevalence in anoxic sediments. *Draconibacterium* and *Ancylomarina* encode dissimilatory nitrate reductases (*narGHI*) and sulfur oxidation modules, supporting survival in low-oxygen sediments.

Variability in cofactor metabolism (e.g., *Sunxiuqinia* vs. *Marinifilum*) suggests niche partitioning driven by micronutrient availability. These findings underscore the metabolic diversity within the order *Marinilabiliales* and its ecological implications in marine biogeochemical cycles. Data availability: Completeness values were derived from KEGG module annotations (Supplementary Table S7). Statistical analyses were performed using genus-averaged percentages; standard deviations ranged from ±5% (highly conserved pathways) to ±25% (variable pathways).

### 3.6 Putative polysaccharide degradative capacity

**Figure 5:**
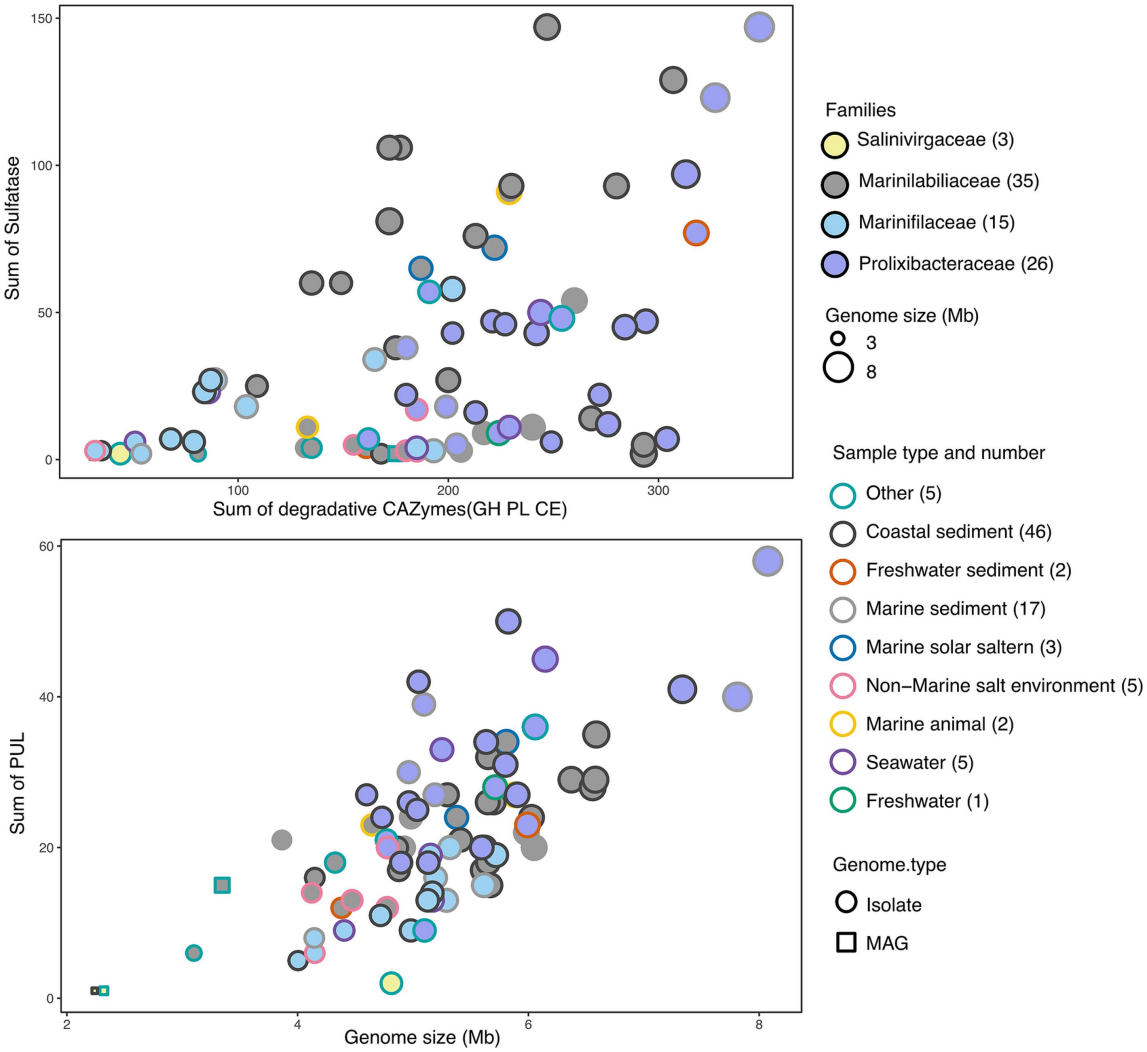
CAZymes versus sulfatase gene frequencies in prominent phyla and families as assessed in 3 MAGs and 77 draft genomes (DGs) from all six sample sources. MAGs were represented by squares and DGs by circles, with border colors representing families and fill colors representing sample types. Circle sizes correspond to genome sizes. Detailed information was provided in Supplementary Table S4.

**Figure 6:**
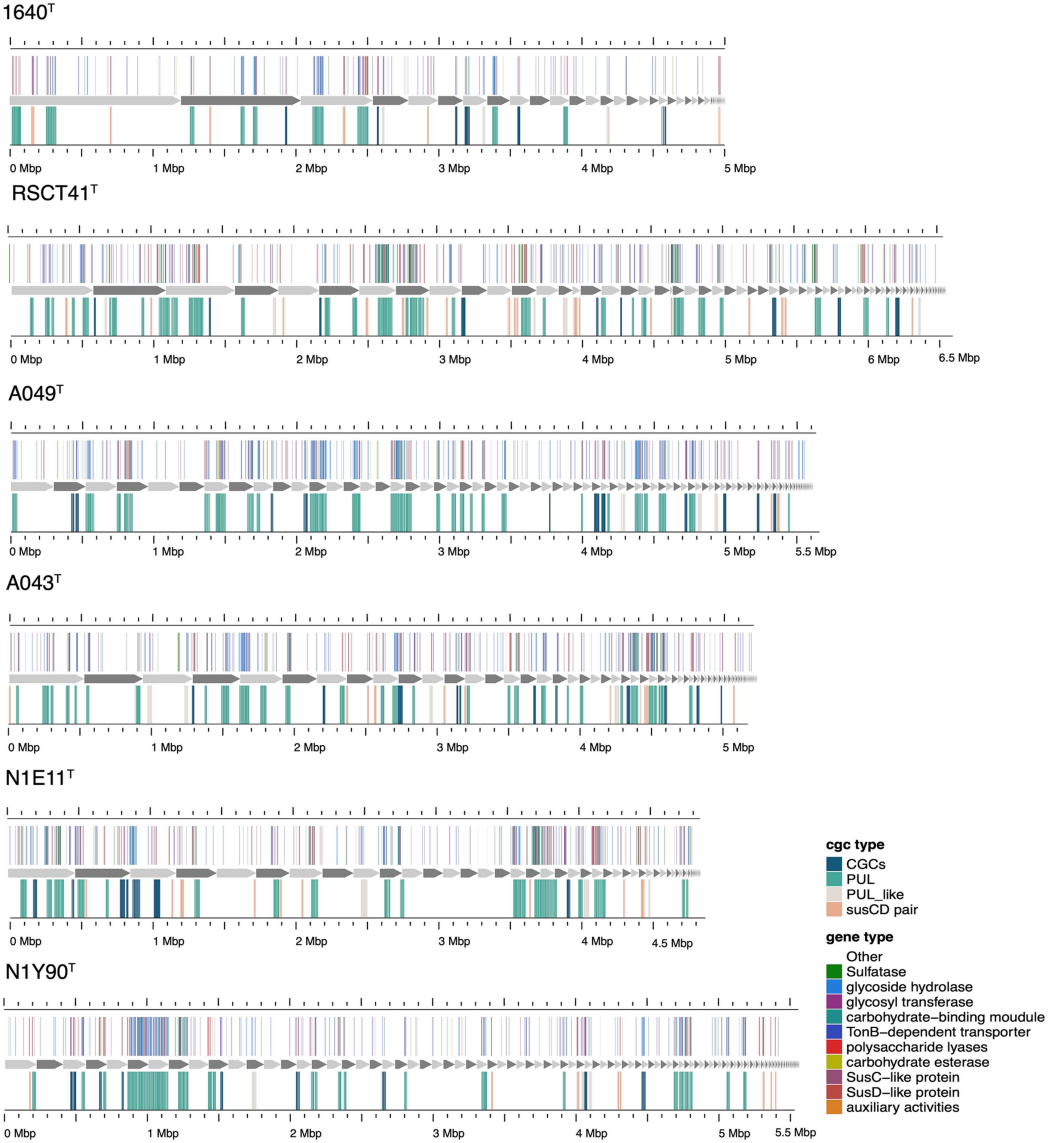
Composition and location of PUL, PUL-like and CGCs on the genome of different strains. Strain names were labeled below each genome graphic. The bands from bottom to top of each strain indicated the type of polysaccharides degradation gene clusters, the composition of gene clusters, and the distribution location of its protein components.

The genes of degradative CAZymes and predicted PULs in each genome indicates the potential for polysaccharide degradation (Supplementary Table S4). Interestingly, the occurrence of CAZymes gene and PULs exhibited an exponential difference among different families (Figure 5). Notably, *Prolixibacteraceae* species were annotated with a higher number of PULs, further underscoring their strong potential for polysaccharide degradation. *Marinilabiliaceae* species were annotated with a second highest number of CAZymes and PULs, followed by *Marinifilaceae*. In contrast, *Salinivirgaceae* species had the lowest counts of both CAZymes and PULs. However, the occurrence of PULs did not show a proportional increase with genome size, suggesting no clear correlation between the two variables. As shown in Figure 5, the distribution of sulfatases across families follows a distinct and consistent pattern, suggesting that their presence is non-random and potentially influenced by environmental or ecological factors. We observed a positive correlation between the numbers of CAZymes, sulfatases, and PULs and the different branches of the phylogenetic tree. However, exceptions to this trend were also evident, such as the case of *M. sediminis* 1640^T^, which, despite being located on a more distantly related branch, was annotated with a relatively higher number of CAZymes, sulfatases, and PULs (see Figure 6).

The six novel species identified in the dataset exhibit unique genomic and functional traits that distinguish them from type strains and other members of their respective genera (Figure 6 and Supplementary Table S4). Within the genus *Carboxylicivirga* (*Marinilabiliaceae*), A049^T^ stands out with a genome size of 5.65 Mb, 26 PUL, and 268 CAZymes (Supplementary Table S8)—placing it among the top enzymatic performers in the genus—yet its moderate sulfatase count (14) suggests a focus on non-sulfated carbohydrate degradation compared to extreme marine specialists like *Carboxylicivirga_marina* (106 sulfatases). Similarly, RSCT41^T^ (6.58 Mb genome, 29 PUL, 172 CAZymes, 81 sulfatases) combines high sulfatase activity with moderate CAZyme diversity, indicating adaptation to sulfated algal polysaccharides in marine sediments. In contrast, N1Y90^T^ (5.60 Mb, 20 PUL, 200 CAZymes, 27 sulfatases) displays a generalist profile, lacking specialization in either sulfatases or CAZymes, potentially occupying a transitional niche between marine and non-marine environments. A043^T^ (5.30 Mb, 27 PUL, 175 CAZymes, 38 sulfatases) (Supplementary Table S9) further highlights *Carboxylicivirga*’s genomic plasticity, with one of the highest PUL counts in the genus but unremarkable sulfatase levels, suggesting a focus on structural carbohydrate breakdown rather than sulfated polymers (Kertesz, 2000).

Collectively, these novel species reveal patterns of functional divergence within their genera. While some, like RSCT41^T^ and A049^T^, emphasize marine adaptation through elevated sulfatase activity, others (e.g., N1Y90^T^) exhibit generalist or niche-specific strategies. Their genomic and enzymatic profiles underscore the importance of integrating taxonomic, metabolic, and environmental data to uncover hidden ecological roles in understudied marine bacteria. These findings highlight the potential for novel species to drive discoveries in carbohydrate metabolism, sulfatase-mediated degradation, and niche partitioning in marine ecosystems.

## 4 Discussion

*Marinifilaceae* within the phylum *Marinilabiliales* in the depolymerization, degradation, and transformation of POM derived from animal and plant detritus *in situ* in the deep sea under anoxic conditions, simultaneously coupled with nitrogen, sulfur, and metal elemental biogeochemical cycling play important roles (Li et al., 2022). Our study identified six novel species within the order *Marinilabiliales*, belonging to the genera *Carboxylicivirga* and *Marinifilum*, which were isolated from marine sediments. A comprehensive phylogenetic, genomic, and phenotypic characterization was subsequently performed. Subsequently, by analyzing genome-wide, we conducted a comparative analysis of the genomic features, habitat, and metabolic potential of *Marinilabiliales* strains.

We then focused on analyzing the similarities and differences of strains across four families in terms of their polysaccharide degradation abilities for different polysaccharide substrates. We observed diverse polysaccharide degradation capacities among *Marinilabiliales* strains, such as for laminarin and alginate. Through comparative analysis, we speculate the presence of new PULs involved in the degradation of laminarin and alginate. Additionally, we observed a positive correlation between the genome size and the number of PULs, suggesting that strains with larger genomes may possess a stronger polysaccharide degradation capacity. This finding was consistent with previous research, which noted a close relationship between genome size and ecological adaptation in marine *Flavobacteriaceae* strains (González et al., 2008; Hehemann et al., 2012). We observed significant diversity in polysaccharide degradation capabilities among the species of *Prolixibacteraceae*, *Marinilabiliaceae* and *Marinifilaceae*, with apparent strong correlation to their taxonomy. Within the same genus, strains exhibited similar PUL profiles and genome sizes. These results suggest that the composition of a species’ PUL repertoire was influenced more by its phylogenetic lineage. The PUL repertoires of the isolates reveal that common and structurally simple polysaccharides, such as laminarin, α-1,4-glucans, and alginate, were frequently targeted by conserved PULs. This suggests that maintaining the enzymatic machinery for degrading these substrates was advantageous for *Marinilabiliales*.

The functional predictions of polysaccharide utilization loci (PULs) in this study primarily rely on sequence similarity-based bioinformatics approaches. While these methods provide valuable insights, their resolution remains inherently lower than that of direct experimental validation. Current limitations in characterizing marine macroalgal polysaccharides may introduce uncertainties in substrate specificity predictions. These conserved PULs serve as priority targets for functional characterization and provide a robust framework for formulating testable hypotheses about their putative polysaccharide substrates. Despite its constraints, this strategy establishes a novel paradigm for prioritizing environmentally significant polysaccharides that were recalcitrant to conventional biochemical analyses. Future investigations should integrate genome-guided metabolomics and enzymatic activity assays to elucidate the ecological contributions of these bacterial lineages and assess their biotechnological potential in marine carbon cycling and algal biomass valorization.

In the present study, we conducted comprehensive polyphasic and genome analyses of six *Marinilabiliales* species isolated from coastal marine sediment samples. By investigating the distribution of the six novel species, we found that *Marinilabiliales* was mainly found in coastal marine sediment environments, small amount existed in non-/low-saline environments, such as freshwater sediment and freshwater. Metabolic pathway analysis showed that the different family’s species were generally the same in terms of carbohydrate metabolism, ATP synthesis, fatty acid metabolism, lipid metabolism and nucleic acid metabolism, but differ significantly in terms of amino acid, cofactor and vitamin synthesis. In taxonomical point of view, the polyphasic characterization based on phenotypic and phylogenetic analyses as well as genome-based comparisons revealed that the strains were representatives of six novel species in the order *Marinilabiliales*. Based on the genotypic, phenotypic and chemotaxonomic data (Supplementary Tables S1–S3), strains RSCT41^T^, N1E11^T^, N1Y90^T^, A049^T^ and A043^T^ represented five novel species of the genus *Carboxylicivirga* and 1640^T^ represented one novel species of the genus *Marinifilum*, for which the name *Carboxylicivirga agarovorans*, *Carboxylicivirga longa*, *Carboxylicivirga caseinilyticus*, *Carboxylicivirga litoralis*, *Carboxylicivirga fragile* and *Marinifilum sediminis* were proposed. The type strains were RSCT41^T^ (=MCCC 1H00314^T^ = KCTC 62601^T^), N1E11^T^ (=MCCC 1H01432^T^ = KCTC 102107^T^), A049^T^ (=MCCC 1H00447^T^ = KCTC 82741^T^), A043^T^ (=MCCC 1H00450^T^ = KCTC 82737^T^), N1Y90^T^ (=MCCC 1H00481^T^ = KCTC 72190^T^) and 1640^T^ (=MCCC 1H01311^T^) were isolated from marine sediment collected at Jingzi Port, Weihai, Shandong Province, China (122.12 E, 37.56 N).

## Data Availability

The GenBank accession number for the 16S rRNA gene sequence of strain RSCT41^T^, N1E11^T^, N1Y90^T^, 1640^T^, A049^T^, and A043^T^ were MH202884, PP516525, MW713796, PV474211, MW713797, and MW704288. The draft genome of strain RSCT41^T^, N1E11^T^, N1Y90^T^, 1640^T^, A049^T^, and A043^T^ have been deposited in GenBank under the accession number JBNGPA000000000, JBNGOZ000000000, JBNGOY000000000, CANMCX000000000, JBNGOX000000000, and JBNGOW00000000.

## Footnotes

^1^ https://lpsn.dsmz.de/

^2^ https://github.com/tseemann/barrnap

^3^ http://www.ezbiocloud.net/eztaxon

^4^ https://archive.jgi.doe.gov/data-and-tools/software-tools/bbtools/
