# Supplementary material for: Six new bacterial species of Marinilabiliales isolated from the marine coastal sediment and reclassified Ancylomarina and Labilibaculum as Marinifilum comb. nov. based on the genome analysis
Source: Front Microbiol. 2025 Jul 23;16:1634775. doi: 10.3389/fmicb.2025.1634775 (PMC12325347; doi:10.3389/fmicb.2025.1634775)
Supplement: Supplementary file 1 [file Data_Sheet_1.docx]

Supplementary Material

Six new bacterial species of *Marinilabiliales* isolated from the marine coastal sediment and reclassified *Ancylomarina* and *Labilibaculum* as *Marinifilum* comb. nov. based on the genome analysis

**Han-Zhe Zhang^1, 2^, Jin-Hao Teng^1, 2^, Hao-Yu Zhou^1, 2^, De-Chen Lu^1^* and Zong-Jun Du^1^***

^1^Marine College, Shandong University, Weihai, Shandong, 264209, China

^2^Joint Science College, Shandong University, Weihai, Shandong, 264209, China

*** Correspondence:**

Zong-Jun Du, Email: duzongjun@sdu.edu.cn

De-Chen Lu, Email: DechenLu@hotmail.com

Telephone and fax number: +86-631-5688303

# Supplementary Data

## Genomic comparison within order of *Marinilabiliales*

This study presents a genus-level comparative analysis of genomic features across 79 strains of order *Marinilabiliales*, focusing on GC content, genome size, completeness, contamination, gene count, and coding density. Data were grouped by genus to identify trends linked to environmental niches. Key findings include significant variations in genome size (3.34–8.08 Mb) and coding density (0.82–0.92), with marine sediment-associated genera exhibiting larger genomes and higher coding efficiency. Genera such as *Carboxylicivirga* and *Saccharicrinis* showed adaptations to complex carbohydrate degradation in anaerobic environments. These results highlight the ecological diversification of the order *Marinilabiliales* through genomic plasticity. *Puteibacter* (8.08 Mb), *Marinifilum* (6.59 Mb), and *Carboxylicivirga* (5.65–6.58 Mb) had larger genomes compared to *Ancylomarina* (4.00–4.40 Mb). Marine sediment genera (*Draconibacterium*, *Saccharicrinis*) averaged 5.8 Mb, while non-marine genera (*Ancylomarina*) were smaller (4.2 Mb). GC content varied among different genera, ranging from 33.4% in *Saccharicrinis* to 46.9% in *Mangrovibacterium*. *Carboxylicivirga* (15 strains), *Saccharicrinis* (8 strains), and *Marinifilum* (6 strains) were predominantly coastal. *Draconibacterium* (6 strains) and *Labilibaculum* (4 strains) thrived in marine sediments with moderate GC content (35.4–40.9%) and large genomes (5.1–7.8 Mb). *Ancylomarina* commonly found in non-marine saline environments, possessed compact genomes (4.0–4.4 Mb) and lower coding densities (0.84–0.87). In contrast, *Geofilum*, associated with deep sub-seafloor sediments, exhibited higher GC contents (44.8–48.9%). These genomic traits suggest distinct evolutionary trajectories between non-marine and marine sediment-associated taxa.

**Description of** ***Carboxylicivirga agarovorans* sp. nov.**

*Carboxylicivirga agarovorans* (a.ga.ro.vo.rans. N.L. neut. n. *agarum*, agar, algal polysaccharide; L. inf. v. vorare, to devour, to digest; N.L. part. adj. *agarovorans*, agar-digesting).

Colonies are yellow-pigmented, circular with entire edges, smooth and 0.5-1.0 mm in diameter after 60 h of incubation at 33 °C on MA. This Gram-stain-negative bacterium displays filamentous cells measuring 0.3–0.4 µm in width and 16–30 µm in length. Growth occurs at 15–37°C (optimum 30–33°C, with 33°C as the optimal point), pH 6.5–8.5 (optimum pH 7.5), and in the presence of 0.5–4.5% (w/v) NaCl (optimum 2–3%). The strain hydrolyzes starch and DNA but does not hydrolyze Tween 20, 40, 60, 80, cellulose, gelatin, or casein. Agar hydrolysis is observed, while oxidase, catalase, and nitrate reduction activities are absent. Cells showed strong positive reactions for alkaline phosphatase, esterase (C4), esterase lipase (C8), trypsin, α-chymotrypsin, and acid phosphatase. Additionally, weak activities (denoted as “w”) were observed for leucine arylamidase, naphthol-AS-BI-phosphohydrolase, and β-glucosidase, indicating low but detectable enzymatic potential in these functions. No activity was detected for lipase (C14), valine arylamidase, cystine arylamidase, and a range of carbohydrate-processing enzymes, including α/β-galactosidase, β-glucuronidase, α/β-glucosidase, N-acetyl-β-glucosaminidase, α-mannosidase, and α-fucosidase. In the API 20NE test, the strain was positive for indole production from L-tryptophan and showed activity for β-glucosidase, β-galactosidase, and gelatin hydrolysis. However, it was negative for nitrate reduction, arginine dihydrolase, urease, and the assimilation of a wide spectrum of carbon sources, including D-glucose, arabinose, mannose, mannitol, N-acetylglucosamine, maltose, potassium gluconate, capric acid, adipic acid, malic acid, citrate, and phenylacetic acid. The major fatty acids were iso-C_15:0_ and anteiso-C_15:0_. Chemotaxonomic analysis identifies menaquinone-7 (MK-7) as the sole respiratory quinone, with polar lipids comprising phosphatidylethanolamine (PE) and an unidentified lipid (L). The genomic DNA G+C content is 39.34 mol%.

The type strain, RSCT41^T^ (= MCCC 1H00314^T^ = KCTC 62601^T^), was isolated from marine sediment, Yantai, China (37°33'38.7"N, 121°17'7.6"E). The 16S rRNA gene sequence is deposited in GenBank under accession number MH202884, and the whole-genome sequence is available under accession number JBNGPA000000000.

**Description of *Carboxylicivirga longa* sp. nov.**

*Carboxylicivirga longa* (lon’ga. L. fem. adj. *longa*, long).

Colonies are yellow-pigmented, circular with entire edges, smooth and 0.5-1.0 mm in diameter after 60 h of incubation at 33 °C on MA. Cells are slender, 0.3–0.4 µm in width and 8–20.5 µm in length and Gram-stain-negative. The strain is oxidase- and catalase-positive and capable of reducing nitrate to nitrite. Growth occurs at 12–45°C (optimum 30–33°C), pH 6.5–9.0 (optimum pH 8.0), and in the presence of 0–6% (w/v) NaCl (optimum 2–3%). It hydrolyzes starch, DNA, carboxymethyl cellulose, agar, and Tween 20 but does not hydrolyze casein, gelatin, or Tweens 40, 60, and 80. Metabolism is likely facultatively anaerobic. Cells are positive for alkaline phosphatase, acid phosphatase, and naphthol-AS-BI-phosphohydrolase. Weak enzymatic activities were noted for esterase (C4), esterase lipase (C8), and leucine arylamidase, indicating a limited capacity for lipid and peptide hydrolysis. Notably, N1E11 showed strong activity for a suite of carbohydrate-degrading enzymes, including α-galactosidase, β-glucuronidase, α-glucosidase, β-glucosidase, N-acetyl-β-glucosaminidase, and α-fucosidase, suggesting a broad ability to process structurally diverse polysaccharides and oligosaccharides. In contrast, the API 20NE results revealed a restricted metabolic range. The strain was negative for nitrate reduction, L-tryptophan degradation, urease, arginine dihydrolase, and gelatin hydrolysis. It failed to assimilate common sugars and carbon sources such as glucose, arabinose, mannose, mannitol, N-acetyl-glucosamine, maltose, and organic acids including gluconate, capric, adipic, malic, and phenylacetic acid. However, β-glucosidase and β-galactosidase activities were detected, consistent with ZYM assay findings, highlighting a preserved hydrolytic potential toward β-linked glycosides even in the absence of broader substrate utilization. The chemotaxonomic profile includes major fatty acids iso-C_15:0_ and anteiso-C_15:0_, with menaquinone-7 (MK-7) as the predominant respiratory quinone. Polar lipids comprise phosphatidylethanolamine and three unidentified lipids. The genomic DNA G+C content is 43.16 mol%.

The type strain, N1E11^T^ (= MCCC 1H01432^T^ = KCTC 102107^T^), was isolated from marine sediment collected at Jingzi Port, Weihai, China (37°33'36.0"N, 122°07'12.0"E). The 16S rRNA gene sequence is deposited in GenBank under accession number PP516525, and the whole-genome sequence is available under accession number JBNGOZ000000000.

**Description of *Carboxylicivirga caseinilyticus* sp. nov.**

*Carboxylicivirga caseinilyticus* (ca.se.i.ni.ly’ti.cus. N.L. neut. n. *caseinum*, casein; N.L. masc. adj. *lyticus*, able to loosen, able to dissolve; from Gr. masc. adj. *lytikos*, dissolving; N.L. masc. adj. *caseinilyticus*, casein-dissolvin).

Cells are a facultatively anaerobic, Gram-stain-negative bacterium with filamentous cells measuring 0.3–0.4 µm in width and 4.6–21.5 µm in length (Fig. 3.2). Colonies grow optimally at 37°C (range 25–40°C), pH 7.0 (range 6.5–8.0), and 3% (w/v) NaCl (range 1–4%). The strain is oxidase-positive and catalase-negative, capable of reducing nitrate to nitrite. It hydrolyzes starch and casein but does not hydrolyze DNA, carboxymethyl cellulose, agar, gelatin, or Tweens 20, 40, 60, and 80. It tested positive for alkaline phosphatase, esterase (C4), esterase lipase (C8), lipase (C14), leucine arylamidase, valine arylamidase, cystine arylamidase, trypsin, α-chymotrypsin, acid phosphatase, naphthol-AS-BI-phosphohydrolase, α-glucosidase, β-glucosidase, and N-acetyl-β-glucosaminidase. No activity was detected for α-galactosidase, β-galactosidase, β-glucuronidase, α-mannosidase, or α-fucosidase, suggesting a limited ability to hydrolyze certain glycosidic linkages. In the API 20NE system, the strain tested positive for β-glucosidase and β-galactosidase activities, as well as for gelatin hydrolysis. However, it showed negative results for nitrate reduction, indole production from L-tryptophan, arginine dihydrolase, urease, citrate utilization, and the assimilation of various carbohydrates and organic acids, including D-glucose, arabinose, mannose, mannitol, N-acetylglucosamine, maltose, potassium gluconate, capric acid, adipic acid, malic acid, and phenylacetic acid. The chemotaxonomic profile includes major fatty acids iso-C_15:0_, anteiso-C_15:0_, iso-C_15:0_ 3-OH, and iso-C_17:0_ 3-OH, with menaquinone-7 (MK-7) as the predominant respiratory quinone. Polar lipids consist of phosphatidylethanolamine, cardiolipin, and four unidentified lipids. The genomic DNA G+C content is 36.13 mol%.

The type strain, A049^T^ (= MCCC 1H00447^T^ = KCTC 82741^T^), was isolated from marine sediment collected at Jingzi Port, Weihai, Shandong Province, China (37°33'36.0"N, 122°07'12.0"E). The 16S rRNA gene sequence is deposited in GenBank under accession number MW713797, and the whole-genome sequence is available under accession number JBNGOX000000000.

**Description of *Carboxylicivirga litoralis* sp. nov.**

*Carboxylicivirga litoralis* li.to.ra’lis. L. fem. adj. litoralis, of the shore.

Cells are Gram-stain-negative bacterium with rod-shaped cells measuring 0.6 µm in width and 9.2 µm in length. The strain grows at 20–40°C (optimum 37°C), pH 6.5–8.0 (optimum pH 7.0), and in the presence of 2–5% (w/v) NaCl (optimum 3%). It is oxidase- and catalase-positive but unable to reduce nitrate. Hydrolytic activities are positive for Tween 20, Tween 40, starch, and casein, whereas hydrolysis of Tween 60, Tween 80, cellulose, DNA, agar, and gelatin is negative. Cells are positive for alkaline phosphatase, esterase (C4), esterase lipase (C8), leucine arylamidase, valine arylamidase, trypsin, α-chymotrypsin, acid phosphatase, naphthol-AS-BI-phosphohydrolase, α-glucosidase, β-glucosidase, and N-acetyl-β-glucosaminidase. In contrast, it lacked detectable activity for lipase (C14), cystine arylamidase, α- and β-galactosidase, β-glucuronidase, α-mannosidase, and α-fucosidase, suggesting a selective enzymatic repertoire focused on glycoside and peptide bond cleavage. In the API 20NE system, cells tested positive for indole production via L-tryptophan degradation, β-glucosidase, β-galactosidase, and gelatin hydrolysis. However, the strain showed negative results for nitrate reduction, arginine dihydrolase, urease activity, and the assimilation of a broad panel of carbohydrates and organic acids, including D-glucose, arabinose, mannose, mannitol, N-acetylglucosamine, maltose, potassium gluconate, capric acid, adipic acid, malic acid, citrate, and phenylacetic acid. The chemotaxonomic characteristics include menaquinone-7 (MK-7) as the sole respiratory quinone and polar lipids comprising phosphatidylethanolamine (PE), diphosphatidylglycerol (DPG), and an unidentified lipid (L). The genomic DNA G+C content is 39.34 mol%.

The type strain, A043^T^ (= MCCC 1H00450^T^ = KCTC 82737^T^), was isolated from marine sediment collected at Jingzi Port, Weihai, Shandong Province, China (37°33'36.0"N, 122°07'12.0"E). The 16S rRNA gene sequence is deposited in GenBank under accession number MW704288, and the whole-genome sequence is available under accession number JBNGOW000000000.

**Description of *Carboxylicivirga fragile***

*Carboxylicivirga fragile* (fra’gi.le. L. neut. adj. *fragile*, fragile)

Cells are a facultatively anaerobic, Gram-stain-negative bacterium with filamentous cells measuring 0.3–0.4 µm in width and 8.5–30.5 µm in length. The strain is oxidase-positive and catalase-negative, capable of reducing nitrate to nitrite. Growth occurs at 20–33°C (optimum 28°C), pH 6.5–8.0 (optimum pH 7.0), and in the presence of 0–7% (w/v) NaCl (optimum 2%). It hydrolyzes starch, casein, and Tweens 20, 40, 60, and 80 but does not hydrolyze carboxymethyl cellulose, DNA, agar, or gelatin. The strain exhibits alkaline phosphatase, leucine arylamidase, valine arylamidase, cystine arylamidase, trypsin, α-chymotrypsin, acid phosphatase, naphthol-AS-BI-phosphohydrolase, and N-acetyl-β-glucosaminidase activities in the API ZYM assay, while showing no detectable activity for esterase (C4), esterase lipase (C8), lipase (C14), α-galactosidase, β-galactosidase, β-glucuronidase, α-glucosidase, β-glucosidase, α-mannosidase, or α-fucosidase. In the API 20NE system, it tests positive for indole production (via L-tryptophan degradation), β-glucosidase, and β-galactosidase, but negative for nitrate reduction, arginine dihydrolase, urease, gelatin hydrolysis, citrate utilization, and assimilation of D-glucose, arabinose, mannose, mannitol, N-acetylglucosamine, maltose, potassium gluconate, capric acid, adipic acid, malic acid, or phenylacetic acid. Carbon source utilization assays confirm the absence of metabolic activity toward these substrates, highlighting a restricted substrate spectrum. Notably, the strain demonstrates hydrolytic capabilities toward β-glucosides and β-galactosides despite its limited carbohydrate assimilation profile. The chemotaxonomic profile includes major fatty acids iso-C_15:0_, anteiso-C_15:0_, C_15:1_ *ω6c*, iso-C_15:0_ 3-OH, and iso-C_16:0_ 3-OH, with menaquinone-7 (MK-7) as the predominant respiratory quinone. Polar lipids consist of phosphatidylethanolamine, an unidentified aminolipid, and three unidentified lipids. The genomic DNA G+C content is 36.96 mol%.

The type strain, N1Y90^T^ (= MCCC 1H00481^T^ = KCTC 72190^T^), was isolated from marine sediment collected at Jingzi Port, Weihai, China (37°33'36.0"N, 122°07'12.0"E). The 16S rRNA gene sequence is deposited in GenBank under accession number MW713796, and the whole-genome sequence is available under accession number JBNGOY000000000.

**Description of *Marinifilum sediminis* sp. nov.**

*Marinifilum sediminis* (se.di’mi.nis. L. gen. neut. n. *sediminis*, of sediment)

This Gram-stain-negative bacterium grows at 25–40°C (optimum 28°C), pH 5.5–9.0 (optimum pH 7–8.5), and in the presence of 1–8% (w/v) NaCl (optimum 2–3%). It is oxidase-positive but catalase-negative, with no observed nitrate reduction activity. The strain hydrolyzes Tween 20, Tween 60, Tween 80, and cellulose, but does not hydrolyze starch, agar, or casein. Gelatin hydrolysis and DNA hydrolysis were not detected. In the API ZYM assay, it demonstrated strong activities for alkaline phosphatase, esterase (C4), esterase lipase (C8), acid phosphatase, α-glucosidase and β-glucosidase, suggesting robust capabilities for phosphoester and lipid hydrolysis. Weak activity was also observed for lipase (C14) and β-galactosidase. Among proteolytic enzymes, leucine arylamidase showed strong activity, while valine arylamidase and cystine arylamidase exhibited weak activity; trypsin and α-chymotrypsin were inactive. However, other glycosidases, such as α-galactosidase, β-glucuronidase, N-acetyl-β-glucosaminidase, α-mannosidase, and α-fucosidase, were not detected, indicating a relatively narrow sugar-degrading enzyme profile. In the API 20NE assay, strain 1640 displayed weak nitrate reduction and urease activities, alongside positive β-glucosidase activity. It did not exhibit gelatin hydrolysis or L-tryptophan degradation. The strain failed to assimilate common monosaccharides, sugar alcohols, and organic acids, including glucose, arabinose, mannose, mannitol, N-acetyl-glucosamine, maltose, and various carboxylic acids. Chemotaxonomic analysis identifies menaquinone-7 (MK-7) as the predominant respiratory quinone and polar lipids including diphosphatidylglycerol (DPG) and phosphatidylethanolamine (PE). The genomic DNA G+C content is 34.49 mol%.

The type strain, 1640^T^ (= MCCC 1H01311^T^), was isolated from marine sediment collected at Jingzi Port, Weihai, Shandong Province, China (37°33'36.0"N, 122°07'12.0"E). The 16S rRNA gene sequence is deposited in GenBank under accession number PV474211, and the whole-genome sequence is available under accession number CANMCX000000000.

**Description of *Marinifilum sediment* comb. nov.**

***Marinifilum sediment*** (se.di.men’ti. L. gen. neut. n. *sedimenti*, from the sediment, referring to the habitats of the type strain; N.L. gen. masc. n. sedimenti retained from the basonym to denote ecological origin).

**Basonym**: [*Fulvivirga*] *sediment* Zhang et al. 2021 [GenBank MW391774; Zhang et al., Int J Syst Evol Microbiol 71: 004992].

The polar lipid profile of Marinifilum sedimenti 1062ᵀ comprises phosphatidylethanolamine (PE), phosphatidylglycerol (PG), diphosphatidylglycerol (DPG), and an unidentified aminolipid, consistent with the lipid composition observed in other *Marinifilum* species. The major fatty acids (>5%) include iso-C₁₅_:_₀ (39.6%), iso-C₁₇_:_₀ 3-OH (14.2%), iso-C₁₅_:_₁ *ω6c*/G (6.0%), iso-C₁₅_:_₀ 3-OH (5.3%), and Summed Feature 3 (C₁₆_:_₁ ω7c/C₁₆_:_₁ *ω6c*) (6.1%), reflecting a predominance of branched-chain and unsaturated fatty acids typical of marine heterotrophic bacteria. The sole respiratory quinone is menaquinone-7 (MK-7), a trait shared with closely related *Marinifilum* taxa. The DNA G+C content of the type strain is 45.1 mol%, aligning with the genomic stability observed in the genus.

**Type Strain**: 1062ᵀ (= KCTC 72868ᵀ = MCCC 1H00499ᵀ), isolated from tidal zone sediment of Xiaoshi Island, Weihai, Shandong Province, China (122°00′58″E, 37°31′36″N).

**GenBank Accessions**: MW391774 (16S rRNA gene), JAIXNE000000000 (genome).

***Marinifilum salipaludis* comb. nov.**

***Marinifilum salipaludis*** (sa.li.pa.lu′dis. L. n. sal, salt; L. gen. n. paludis, of a swamp; N.L. gen. masc. n. salipaludis, referring to the salt marsh habitat of the type strain).

**Basonym**: [Ancylomarina] salipaludis SHSM-M15ᵀ Kim et al., 2020.

The polar lipid profile of Marinifilum salipaludis SHSM-M15ᵀ includes phosphatidylethanolamine (PE) as the dominant component, consistent with the lipid composition observed in other Marinifilum species. The major fatty acids (>10% of total) are iso-C₁₅_:_₀, iso-C₁₅_:_₀ 3-OH, and anteiso-C₁₅_:_₀, reflecting a predominance of branched-chain fatty acids characteristic of marine and halophilic bacteria. The sole respiratory quinone is menaquinone-7 (MK-7), a trait shared with closely related Marinifilum taxa. The DNA G+C content of the type strain is 36.6 mol%, aligning with the genomic diversity observed within the genus.

**Type Strain**: SHSM-M15ᵀ (= KACC 19862ᵀ = NBRC 113749ᵀ), isolated from a salt marsh at Siheung, Yellow Sea, Republic of Korea. **GenBank Accessions**: MK577405 (16S rRNA gene), SAXA00000000 (genome).

***Marinifilum subtilis* comb. nov.**

***Marinifilum subtilis*** (sub′ti.lis. L. fem. adj. subtilis, slender, referring to the slender cells of the type strain; N.L. fem. n. subtilis retained from the basonym to denote morphological traits).

**Basonym**: [Ancylomarina] subtilis FA102ᵀ Wu et al. 2016.

The polar lipid profile of Marinifilum subtilis FA102ᵀ includes phosphatidylethanolamine (PE) as the dominant component, along with two unidentified aminolipids and minor amounts of unidentified phospholipids, phosphoaminolipids, and neutral lipids. This composition aligns with the lipid patterns observed in other Marinifilum species. The major fatty acids (>5%) are iso-C₁₅_:_₀ (34.2%), iso-C₁₅_:_₀ 3-OH (18.5%), iso-C₁₅_:_₁F (12.1%), iso-C₁₃_:_₀ (9.8%), and anteiso-C₁₅_:_₀ (7.3%), reflecting a predominance of branched-chain and hydroxylated fatty acids typical of marine heterotrophic bacteria. The sole respiratory quinone is menaquinone-7 (MK-7), a trait shared with closely related Marinifilum taxa. The DNA G+C content of the type strain is 36.5 mol%, consistent with the genomic diversity observed in the genus.

**Type Strain**: FA102ᵀ (= KCTC 42257ᵀ = DSM 28825ᵀ = CICC 10902ᵀ), isolated from marine sediment off the coast of Weihai, China. **GenBank Accessions**: MK577406 (16S rRNA gene), SAXA00000001 (genome).

***Marinifilum antarcticum* comb. nov.**

***Marinifilum antarcticum*** (ant.arc’ti.cum. L. neut. adj. antarcticum, southern, belonging to Antarctica; N.L. neut. adj. antarcticum retained from the basonym to denote geographic origin).

**Basonym**: [Labilibaculum] antarcticum SPP2ᵀ Watanabe et al. 2020.

The polar lipid profile of Marinifilum antarcticum SPP2ᵀ includes phosphatidylethanolamine (PE), phospholipids, an aminolipid, a phosphoaminolipid, and three unidentified lipids, consistent with the lipid composition observed in other Marinifilum species. The major fatty acids (>5%) are anteiso-C₁₅_:_₀ (34.2%) and iso-C₁₅_:_₀ (18.5%), reflecting a predominance of branched-chain fatty acids typical of cold-adapted marine bacteria. The sole respiratory quinone is menaquinone-7 (MK-7), a trait shared with closely related Marinifilum taxa. The DNA G+C content of the type strain is 36.0 mol%, aligning with the genomic diversity observed in the genus.

**Type Strain**: SPP2ᵀ (= NBRC 111151ᵀ = CECT 9460ᵀ), isolated from marine sediment in Antarctica. **GenBank Accessions**: LC085518 (16S rRNA gene), AP018042 (genome).

***Marinifilum filiforme*** **comb. nov.**

***Marinifilum filiforme*** (fi.li.for'me. L. n. filum, thread; L. suff. -forme, shaped; N.L. neut. adj. filiforme, thread-shaped, referring to filamentous cell morphology).

**Basonym**: [Labilibaculum] filiforme 59.16Bᵀ Vandieken et al. 2019.

Cells are Gram-stain-negative rods (0.5–0.8 × 2.2–3.0 µm), forming filaments under certain growth conditions. Psychrotolerant, with growth at 4–25 °C (optimum 20–25 °C) and pH 6.5–8.0 (optimum 7.4). Tolerates 0.05–6.5% NaCl (optimum 0.5–1.0%). Ferments glucose, cellobiose, N-acetylglucosamine, and, in some strains, arginine. Reduces Fe (III) oxides but not M n(IV) under microaerophilic conditions. Gliding motility is observed. Colonies on marine agar are beige, circular, and 0.8–1.2 mm in diameter after 2–3 days at 25 °C.

**Type Strain**: 59.16Bᵀ (= DSM 101180ᵀ = JCM 31101ᵀ), isolated from subsurface marine sediment of the Little Belt, Baltic Sea. **GenBank Accessions**: CP021095 (genome), LC085519 (16S rRNA gene).

***Marinifilum manganireducens* comb. nov.**

***Marinifilum manganireducens*** (man.ga.ni re.du'cens. N.L. n. manganum, manganese; L. part. adj. reducens, reducing; N.L. part. adj. manganireducens, referring to the ability to reduce Mn (IV) oxides).

**Basonym**: [Labilibaculum] manganireducens 59.10-2Mᵀ Vandieken et al. 2019.

Cells are Gram-stain-negative rods (0.6–0.8 × 0.9–3.8 µm), psychrotolerant, with growth at 4–30 °C (optimum 30 °C) and pH 5.9–8.0 (optimum 7.5–8.0). Tolerates 0.5–6.5% NaCl (optimum 1–2.5%). Ferments fructose, glucose, arabinose, cellobiose, N-acetylglucosamine, and rhamnose. Reduces Fe (III) and Mn (IV) oxides as electron acceptors under microaerophilic conditions. Gliding motility with occasional flips is observed. Colonies on marine agar are beige, circular, and 1–1.5 mm in diameter after 2–3 days at 28 °C.

**Type Strain**: 59.10-2Mᵀ (= DSM 102944ᵀ = JCM 31100ᵀ), isolated from subsurface marine sediment of the Little Belt, Baltic Sea. **GenBank Accessions**: CP021094 (genome), LC085518 (16S rRNA gene).

# Supplementary Figures


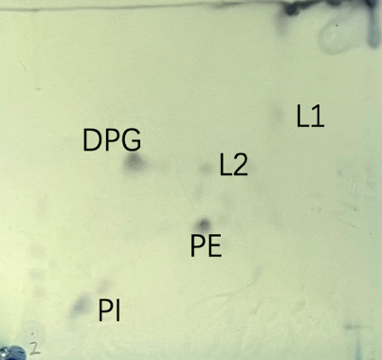

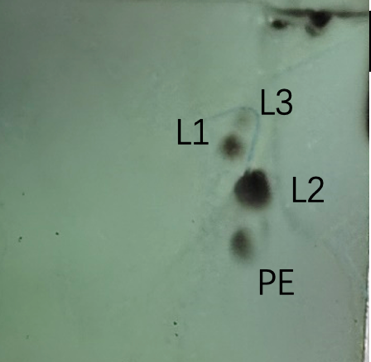

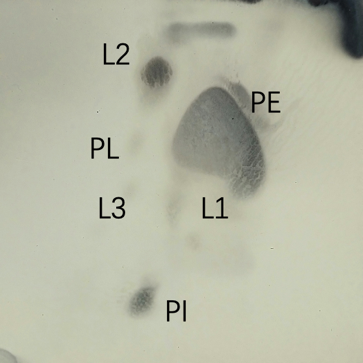
 RSCT41^T^ N1E11^T^ 1640^T^

**Second dimension**

**First dimension**


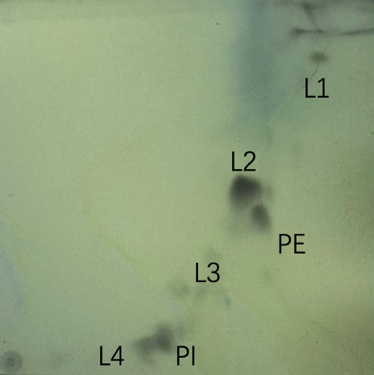
**
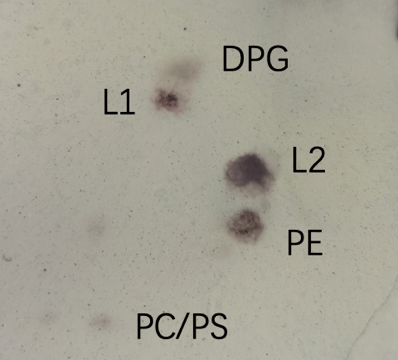
**
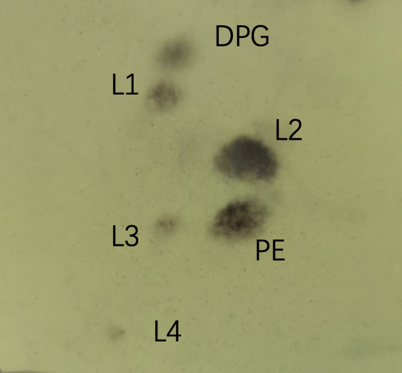
N1Y90^T^ A049^T^ A043^T^

**Second dimension**

**First dimension**

**Supplementary Figure S1.** Two-dimensional TLC plate images of lipids of strain RSCT41^T^, N1E11^T^, N1Y90^T^, 1640^T^, A049^T^ and A043^T^. The plate is sprayed with 5% molybdatophosphoric acid to show all lipids. L1-6, Lipid; PL, Phospholipid; PE, Phosphatidylethanolamine; PNL, Phosphoaminolipid.

**Supplementary Figure S2.** Ecological distribution of six strains across various habitats, as shown in the radar plots for different strains RSCT41^T^, N1E11^T^, N1Y90^T^, 1640^T^, A049^T^ and A043^T^. Each plot illustrates the relative abundance of the strains across multiple environmental categories, including plant, aquatic freshwater, aquatic marine, aquatic sediment, animal habitats, aquatic unknown, soil, and unknown habitats. The numbers next to each habitat label represent the number of sequences identified within that category. The green shaded area in each radar plot indicates the strain's distribution in that habitat, with the scale ranging from 0 to 1.
